# UBIAD1 suppresses the proliferation of bladder carcinoma cells by regulating H-Ras intracellular trafficking via interaction with the C-terminal domain of H-Ras

**DOI:** 10.1038/s41419-018-1215-4

**Published:** 2018-12-05

**Authors:** Zhiliang Xu, Fengsen Duan, Huiai Lu, Maytham Abdulkadhim Dragh, Yanzhi Xia, Huageng Liang, Ling Hong

**Affiliations:** 1 Department of Genetics and Developmental Biology, College of Life Science and Technology, Huazhong University of Science and Technology, Wuhan, Hubei People’s Republic of China; 2 Department of Urology, Union Hospital, Tongji Medical College, Huazhong University of Science and Technology, Wuhan, Hubei People’s Republic of China

## Abstract

UbiA prenyltransferase domain-containing protein 1 (UBIAD1) plays a key role in biosynthesis of vitamin K_2_ and coenzyme Q10 using geranylgeranyl diphosphate (GGPP). However, the mechanism by which UBIAD1 participates in tumorigenesis remains unknown. This study show that UBIAD1 interacts with H-Ras, retains H-Ras in the Golgi apparatus, prevents H-Ras trafficking from the Golgi apparatus to the plasma membrane, blocks the aberrant activation of Ras/MAPK signaling, and inhibits the proliferation of bladder cancer cells. In addition, GGPP was required to maintain the function of UBIAD1 in regulating the Ras/ERK signaling pathway. A *Drosophila* model was employed to confirm the function of UBIAD1/HEIX in vivo. The activation of Ras/ERK signaling at the plasma membrane induced melanotic masses in *Drosophila* larvae. Our study suggests that UBIAD1 serves as a tumor suppressor in cancer and tentatively reveals the underlying mechanism of melanotic mass formation in *Drosophila*.

## Introduction

Ras proteins are essential components of the intracellular signaling cascades that regulate various fundamental cellular activities such as proliferation, apoptosis, differentiation, and senescence^1,2^. One of the central paradoxes involving Ras signal transduction is how Ras, which acts as a simple binary switch, can generate many different biological outputs^3–5^. The intracellular trafficking cycle of Ras partially accounts for this phenomenon^6^. Ras interacts dynamically with specific microdomains of the plasma membrane and other internal cell membranes. These different membrane microenvironments modulate Ras signal output, highlighting the complex interplay between Ras location and function^7^. For example, Ras signaling in the plasma membrane is rapid and transient, whereas that in the Golgi apparatus is delayed and sustained^8^. Ras activates Raf-MEK-ERK in the plasma membrane and leads to cellular growth^6^. Cellular differentiation is induced when Ras activates Raf or PI3K in the Golgi apparatus^9,10^.

H-Ras, which accounts for all Ras mutations in bladder carcinoma^6^, is also common in thyroid cancer, salivary carcinoma^11^, epithelial myoepithelial cancer and kidney cancer^12^. After being synthesized in cytosolic ribosomes, H-Ras undergoes farnesylation at the cysteine of the C-terminal CVLS motif in the cytosol, followed by proteolysis of the VLS sequence in the endoplasmic reticulum (ER). However, as farnesylation of Ras provides only a weak signal for membrane interaction, another motif in the HVR (hypervariable region) of Ras is required for membrane association. Two palmitoylation groups at the cysteine, adjacent to the farnesylated cysteine of H-Ras, are the second membrane-anchoring signal. Palmitoylation primarily occurs at the Golgi surface, and palmitoylated H-Ras then aggregates to the plasma membrane via a secretory pathway for eventual insertion into the plasma membrane^13^. In addition, palmitoylated H-Ras undergoes dynamic de/re-acylation with depalmitoylation leading to detachment from the plasma membrane to facilitate transfer to the Golgi apparatus^14,15^. Bladder carcinoma with H-Ras mutation^16^ ranks ninth in cancer incidence worldwide^17^. Considering that H-Ras induces melanoma development in mice^18^ and melanotic spots appear in *heix/ubiad1* mutant *Drosophila*^19^, we focused on establishing the relationship between UBIAD1 and H-Ras.

*UBIAD1* (also known as *TERE1*), a cancer suppressor gene in bladder carcinoma^20,21^, encodes a class of UbiA prenyltransferases^22^ and participates in the regulation of cholesterol^23–26^. UBIAD1 is widely expressed in various human tissues^20^ and organelles, including the endothelial reticulum^19,22^, Golgi apparatus^27,28^, and mitochondria^29^. Interestingly, UBIAD1, with both side-chain cleavage and its prenylation activity, is the first enzyme responsible for human vitamin K biosynthesis^22,30^. Moreover, UBIAD1 is a nonmitochondrial CoQ10-forming enzyme with a specific cardiovascular antioxidant function via regulation of eNOS activity in *zebrafish*^27^. Given that UBIAD1 participates in various biological processes, its downregulation or mutation can induce the development of diseases such as cancer^20,31^, Schnyder crystalline corneal dystrophy (SCCD)^32^, Parkinson’s disease^29^, and cardiovascular disease^27^.

Recent research on this tumor suppressor show that UBIAD1 protects the cholesterol biosynthetic enzyme HMG-CoA reductase from degradation^33–35^ and plays an important role in vascular cell calcification^36^. DNA methylation of *ubiad1* has significant negative associations with EGFR/KRAS mutations in lung adenocarcinoma^37^. Furthermore, considering that UBIAD1 is downregulated in bladder and prostate carcinomas, and its overexpression inhibits tumor cell proliferation^21,38^. We previously reported that UBIAD1 knockdown activates the Ras/MAPK signaling pathway^39^.

Here, we report that UBIAD1 interacts with H-Ras, increases the retention of H-Ras in the Golgi apparatus, inhibits the aberrant activation of Ras/ERK signaling at the plasma membrane and consequently suppresses the proliferation of bladder cancer cells.

## Results

### UBIAD1 inhibited the activation of the Ras/MAPK signaling pathway

**Fig. 1:**
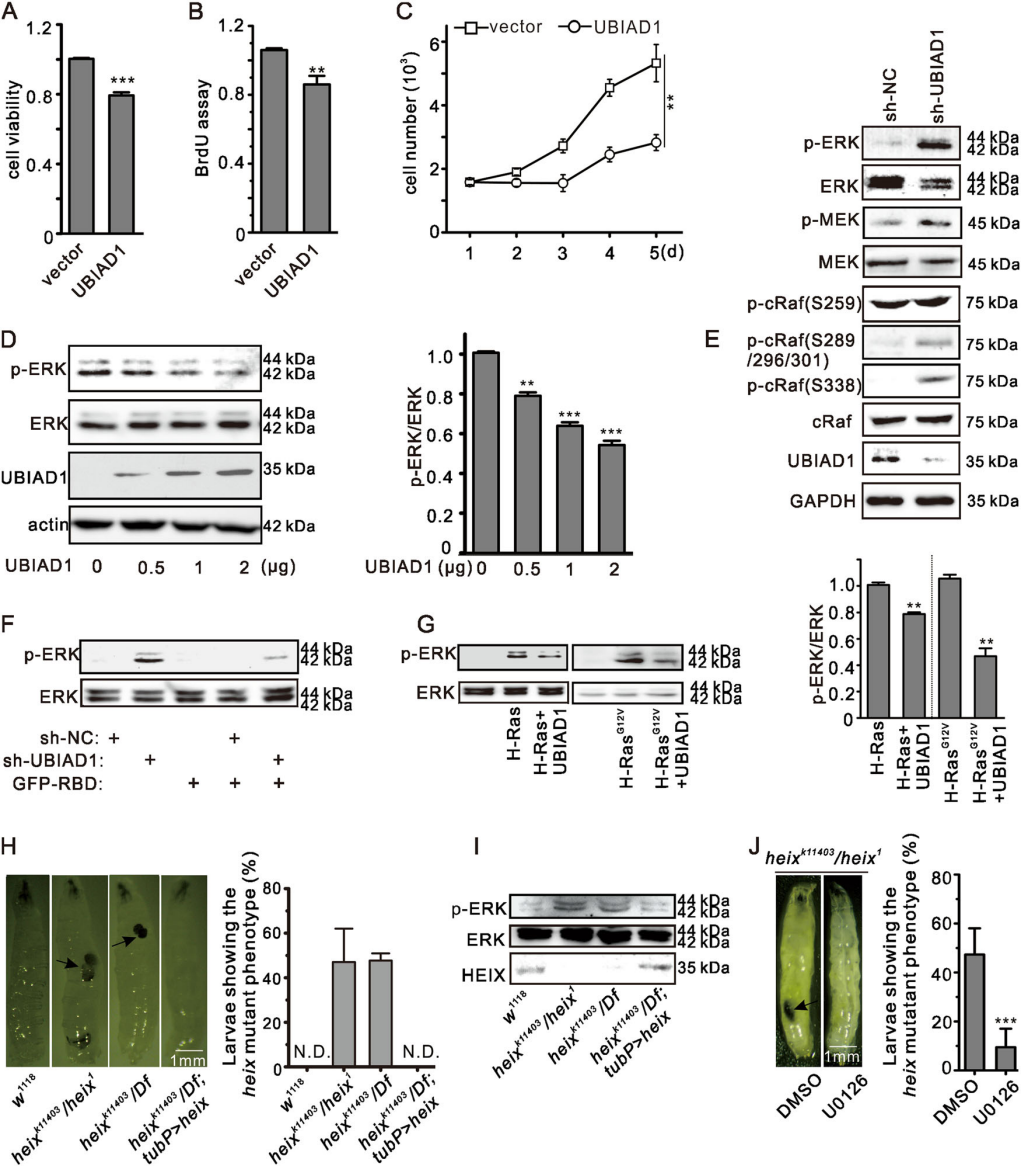
UBIAD1 inhibits the Ras/ERK signaling pathway. **a** UBIAD1 reduced cell viability in T24 bladder cancer cells. T24 cells were transiently transfected with pcDNA3.1-UBIAD1 plasmid. Twenty-four hours after transfection, cell viability was detected by the MTT assay. ***p < 0.001, Student’s *t*-test, *n* = 6 experiments. **b** UBIAD1 inhibited the proliferation of T24 cells. T24 cells were transiently transfected with pcDNA3.1-UBIAD1 plasmid. Twenty-four hours after transfection, cell proliferation was detected using the BrdU cell proliferation ELISA kit. **p < 0.01, Student’s *t*-test, *n* = 3 experiments. **c** UBIAD1 inhibited the growth of T24 cells. T24 cells were transiently transfected with pcDNA3.1-UBIAD1 plasmid. Twenty-four hours after transfection, cell growth was detected by cell counting. **p < 0.01, Student’s *t*-test, *n* = 3 experiments. **d** UBIAD1 inhibits ERK phosphorylation in T24 cells. T24 cells were transfected with increasing amounts of pcDNA3.1-UBIAD1. Twenty-four hours after transfection, total cell lysate was examined by western blotting (WB). The same experiment was repeated at least three times and representative data are shown. This protocol is the same for most immunoblotting analyses throughout the study. The right panel represents the ratio (mean ± SD) from densitometry analyses. **p < 0.01, ***p < 0.001, Student’s *t*-test. **e** The Ras/ERK signaling pathway is activated by UBIAD1 deficiency in HEK293T cells. HEK293T cells were transfected with sh-UBIAD1. Seventy-two hours after transfection, total cell lysate was exposed to antibodies and WB was performed, as indicated. The same experiment was repeated three times. **f** Upregulated p-ERK was abrogated by GFP-RBD. HEK293T cells were transfected with plasmids as indicated. After 72 h of transfection, the total cell lysate was exposed to antibodies and examined by WB. The same experiment was repeated three times. **g** UBIAD1 inhibited H-Ras-induced p-ERK. HEK293T cells were transfected with plasmids as indicated. Twenty-four hours, the total cell lysate was first exposed to antibodies and then examined by WB. The same experiment was repeated three times. The right panel represents the ratio (mean ± SD) from densitometer analyses. **p < 0.01, Student’s *t*-test. **h** The mutation of *heix* (*heix*^*k11403*^*/Df* and *heix*^*k11403*^*/heix*^*1*^) produced melanotic masses in *Drosophila* larvae; *w*^*1118*^ is the wild-type and *heix*^*k11403*^*/Df; tub P* *>* *heix* is the rescue type. Melanotic masses was detected in long larvae following crosses performed for 2 weeks. N.D.: not detected, (*n* = 3 experiments, each with 50 larvae). **i** The mutation of *heix* increased p-ERK in *Drosophila* larvae. Total larvae lysate was exposed to antibodies and examined by WB as indicated in the material and methods. The same experiment was repeated three times. **j** Melanotic masses disappeared under U0126 treatment in the *heix* mutant larvae. Melanotic masses were detected in long larvae following 2 weeks crosses. ***p < 0.001, Student’s *t*-test, (*n* = 3 experiments, each with 50 larvae)

In previous studies, UBIAD1 downregulation has been shown to induce the activation of the Ras/MAPK signaling pathway^39^, and UBIAD1 has inhibited the growth of bladder (Fig. 1a-c)^20^ and prostate cancers^21^. However, the underlying mechanism and relationship between UBIAD1 and Ras/MAPK signaling have not been clearly elucidated. Thus, we examined ERK signaling, following the graded overexpression of UBIAD1 and found dose-dependent inhibition of ERK phosphorylation (p-ERK) in T24 cells (Fig. 1d and Supplementary Fig. S1a, b). To further explore the functional role of UBIAD1 in Ras/ERK signaling, we employed shRNA to knock down endogenous UBIAD1. Phosphorylation of ERK, MEK and c-Raf significantly increased when UBIAD1 was knocked down (Fig. 1e and Supplementary Fig. S1c, d). A rescue assay was performed to confirm the specificity of the silencing effect of UBIAD1-shRNA. Activation of Ras/MAPK signaling by knocking down UBIAD1 was abrogated by UBIAD1 (Supplementary Fig. S1e). In addition, an increase in p-ERK was prevented by the green fluorescence protein-Ras-binding domain (GFP-RBD), which efficiently bound to Ras in the GTP-bound state to competitively inhibit Ras activity (Fig. 1f and Supplementary Fig. S1f). These results indicate that UBIAD1 suppresses Ras activation. UBIAD1 is not expressed in bladder tumors^20^, and H-Ras mutations, which affect MAPK pathways, are associated with bladder carcinoma^40^. Therefore, UBIAD1 function might be related to H-Ras. To verify this hypothesis, HEK293T cells were cotransfected with H-Ras (or H-Ras^G12V^) and UBIAD1. UBIAD1 inhibited both H-Ras-induced and H-Ras^G12V^-induced p-ERK (Fig. 1g), which indicates that UBIAD1 is a negative regulator of H-Ras.

We used *Drosophila* as an animal model to further study and confirm the function of UBIAD1 in vivo. P-ERK levels were increased in *heix* (the homologous gene of *ubiad1) Drosophila* mutants (one P-element allele: *heix*^*k11403*^ and one ethylmethansulfonate allele: *heix*^*1*^). Neither *heix*^*k11403*^ nor *heix*^*1*^ express the HEIX protein^29^. These findings are consistent with a previous study reporting that *heix* regulates expression of *yan*, which is mediated by Ras^19^. Overexpression of the *heix* gene in *heix* mutants decreased phosphorylated ERK levels and led to the subsequent disappearance of melanotic masses (Fig. 1h, i, and Supplementary Fig. S1g). Moreover, melanotic masses in *heix* mutants vanished after U0126 treatment (MEK inhibitor), suggesting that melanotic mass results from abnormal activation of Ras/ERK signaling (Fig. 1j).

### UBIAD1 inhibited H-Ras trafficking from the Golgi apparatus to the plasma membrane

**Fig. 2:**
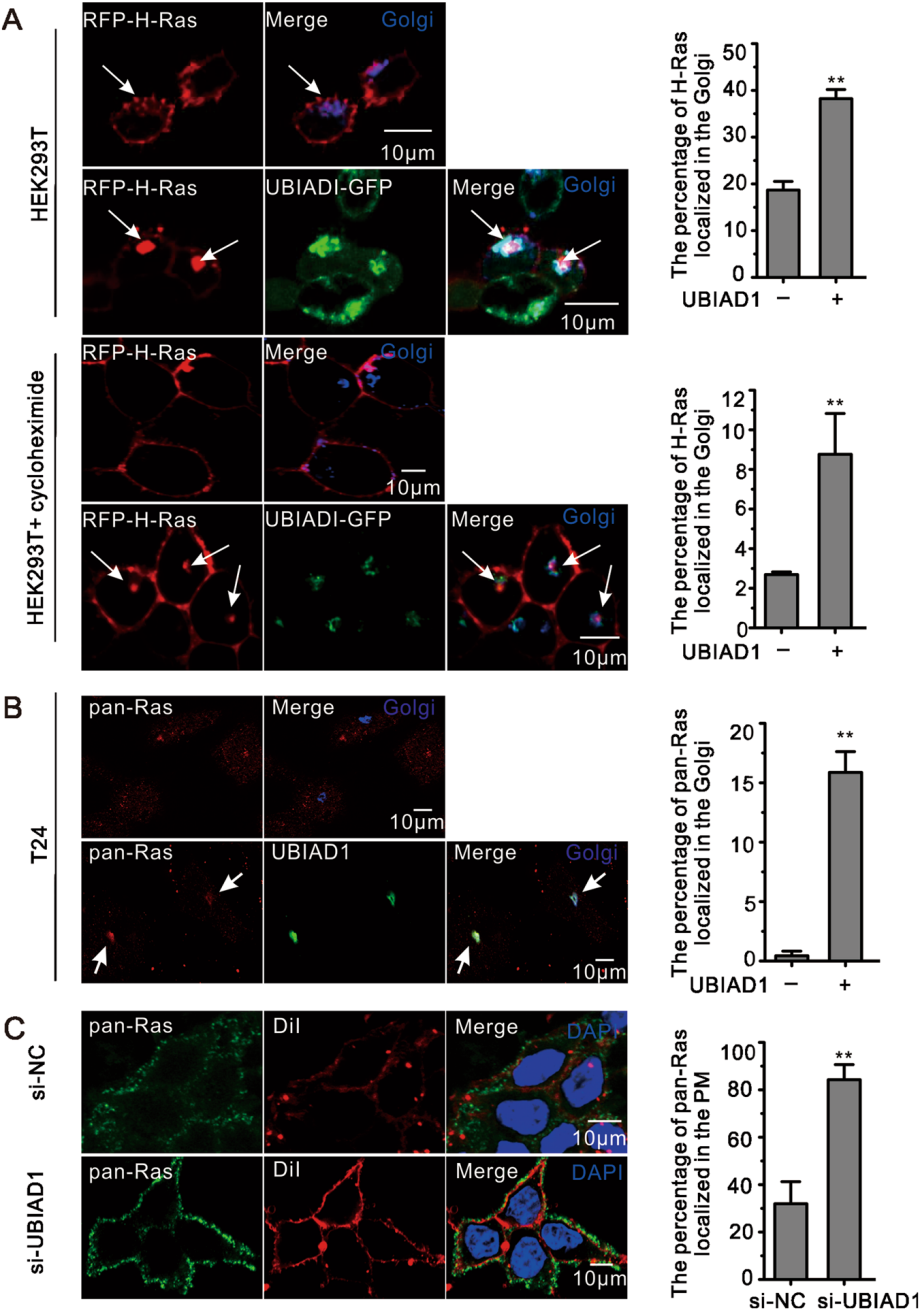
UBIAD1 regulates H-Ras trafficking from the Golgi apparatus to the plasma membrane. **a** UBIAD1 increased H-Ras retention in the Golgi apparatus in HEK293T cells. HEK293T cells were transfected with RFP-H-Ras with or without UBIAD1-GFP, followed by 24 h of culturing with or without cycloheximide for 5 h and confocal analysis. Histograms show the percentage of H-Ras localized in the Golgi. **p < 0.01 compared to control, (*n* = 3 experiments, each with 100 cells). **b** Ectopic expression of UBIAD1 increased the localization of Ras in the Golgi apparatus. T24 cells were transfected with or without UBIAD1-GFP, followed by 24 h of culture, stained with pan-Ras antibody and analyzed by confocal microscopy. Histograms show the percentage of Ras localized in the Golgi. **p < 0.01 compared to control, (*n* = 3 experiments, each with 100 cells). **c** Ras localized in the plasma membrane after knockdown of UBIAD1. HEK293T cells were transfected with sh-UBIAD1, followed by 72 h of culturing, staining with DiI (a maker of membrane), pan-Ras antibody and DAPI and confocal analysis. Histograms show the percentage of Ras localized in the plasma membrane. **p < 0.01 compared to control, (*n* = 3 experiments, each with 100 cells)

Considering that UBIAD1 is a Golgi-localized protein (Supplementary Fig. S2a)^28^ that acts on H-Ras, we investigated whether UBIAD1 could alter the localization of H-Ras in the Golgi apparatus. When H-Ras (or H-Ras^G12V^) was overexpressed in HEK293T cells, H-Ras was widely localized in the plasma membrane with little traces in the Golgi apparatus, which is consistent with previous reports^41,42^. However, when coexpressed with UBIAD1-EGFP in HEK293T and T24 cells, the localization of H-Ras in the Golgi apparatus significantly increased (Fig. 2a and Supplementary Fig. S2b, c). We determined whether overexpression of the protein is responsible for the accumulation of H-Ras in the Golgi apparatus. The results showed that UBIAD1 increased the retention of H-Ras in the Golgi apparatus after treatment with cycloheximide, which blocks protein synthesis (Fig. 2a). UBIAD1 increased endogenous pan-Ras retention in the Golgi apparatus of T24 cells (Fig. 2b). UBIAD1 also promoted the localization of endogenous H-Ras^G12V^ marked with GFP-RBD in the Golgi apparatus of T24 cells (Supplementary Fig. S2d). Alternatively, when UBIAD1 was deficient in HEK293T cells, high amounts of Ras were transported to the plasma membrane (Fig. 2c and Supplementary Fig. S2e). These data indicate that UBIAD1 regulates the localization of H-Ras (or H-Ras^G12V^) in the Golgi apparatus.

**Fig. 3:**
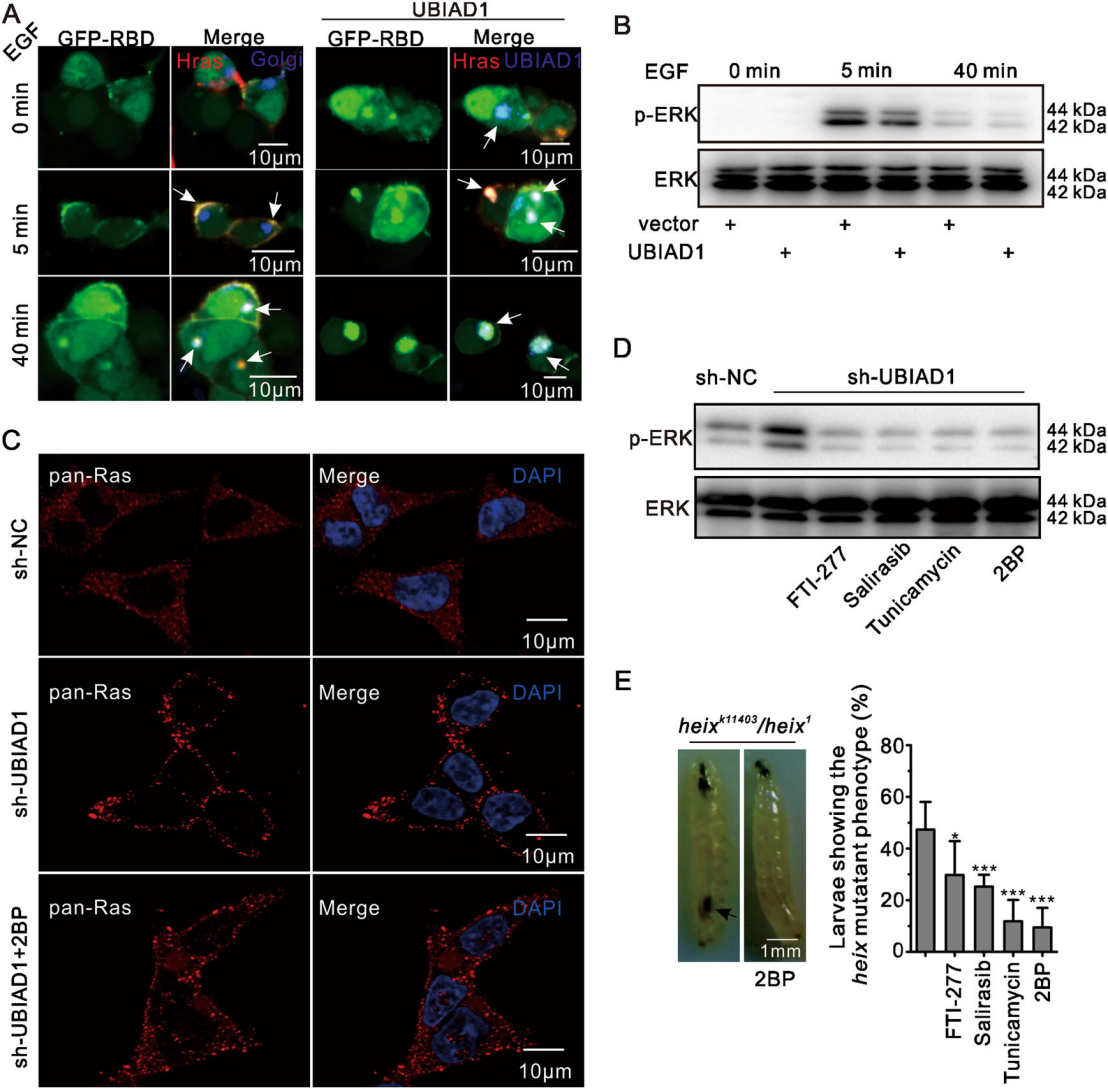
UBIAD1-regulated ERK signaling initiated from the plasma membrane. **a** UBIAD1 changed the localization of H-Ras under short-term (5 min) EGF treatment. HEK293T cells were transfected with plasmids as indicated. After 24 h of transfection, the cells were serum-starved for 6 h before treatment with EGF (100 ng/ml) for different periods of time, followed by confocal analysis, the same experiment was repeated three times. **b** UBIAD1 decreased short-term EGF-induced p-ERK. HEK293T cells were transfected with plasmids as indicated. Twenty-four hours after transfection, the cells were serum-starved for 6 h before treatment with EGF (100 ng/ml) for different periods of time. The total cell lysate was exposed to antibodies and examined by WB as indicated. The same experiment was repeated three times. **c** Ras aggregates in the plasma membrane after knocking down UBIAD1. HEK293T cells were transfected with sh-UBIAD1, followed by 72 h of culture and 24 h of culturing with or without 2BP, staining with pan-Ras antibody and confocal analysis. The same experiment was repeated three times. **d** Ras inhibitors reduced ERK phosphorylation after UBIAD1 knockdown. HEK293T cells were transfected with sh-UBIAD1, followed by 72 h of culture with or without Ras inhibitors (FTI-277, Salirasib, 2BP, tunicamycin). The total cell lysate was exposed to antibodies and WB was performed as indicated. The same experiment was repeated three times. **e** Melanotic masses disappeared under Ras inhibitor treatment in the *heix* mutant larvae. Melanotic masses were detected in long larvae following 2 weeks crosses. *p < 0.05, ***p < 0.001, Student’s *t*-test, (*n* = 3 experiments, each with 50 larvae)

An EGF (Epidermal Growth Factor) assay was performed to analyze the possible connection between the retention of H-Ras in the Golgi apparatus and the decrease in p-ERK. Under unstimulated conditions, GFP-RBD was not associated with any intracellular structures, including the plasma membrane^9^. By contrast, when cells were treated with EGF, GFP-RBD first aggregated in the plasma membrane (Ras activation in the plasma membrane followed by Ras/ERK signaling) and then in the Golgi apparatus^43^. However, when GFP-RBD and H-Ras were coexpressed with UBIAD1, UBIAD1 prevented the transport of H-Ras to the plasma membrane under short-term treatment with EGF (Fig. 3a). UBIAD1 decreased p-ERK levels only under short-term treatment with EGF, indicating that ERK phosphorylation was inhibited by UBIAD1 in the plasma membrane (Fig. 3b). Interestingly, when UBIAD1 was deficient, large amounts of Ras were transported to the plasma membrane, which was abrogated by 2BP (2-bromopalmitate, an inhibitor of palmitoyltransferase, which is necessary for Ras transport to the plasma membrane^44^) (Fig. 3c). Ras inhibitors (FTI-277, Salirasib) and palmitoyltransferase inhibitors (2BP, tunicamycin) prevented ERK phosphorylation in the absence of UBIAD1 (Fig. 3d). This result indicates that the ERK signaling induced by knockdown of UBIAD1 initiated in the plasma membrane.

To confirm that the melanotic mass formation in *heix* mutants was related to Ras signaling, an inhibition assay was performed, fewer traces of melanotic masses were detected in the *heix* mutants when treated with FTI-277, Salirasib, 2BP, tunicamycin (Fig. 3e). These data, combined with cellular experimental results, suggest that large quantities of Ras could be transported to the plasma membrane to activate Ras/ERK signaling. Consequently, the activate Ras signaling induced melanotic masses in *Drosophila*, which is consistent with previous reports^19,45^.

### UBIAD1 interacted with H-Ras in the Golgi apparatus

**Fig. 4:**
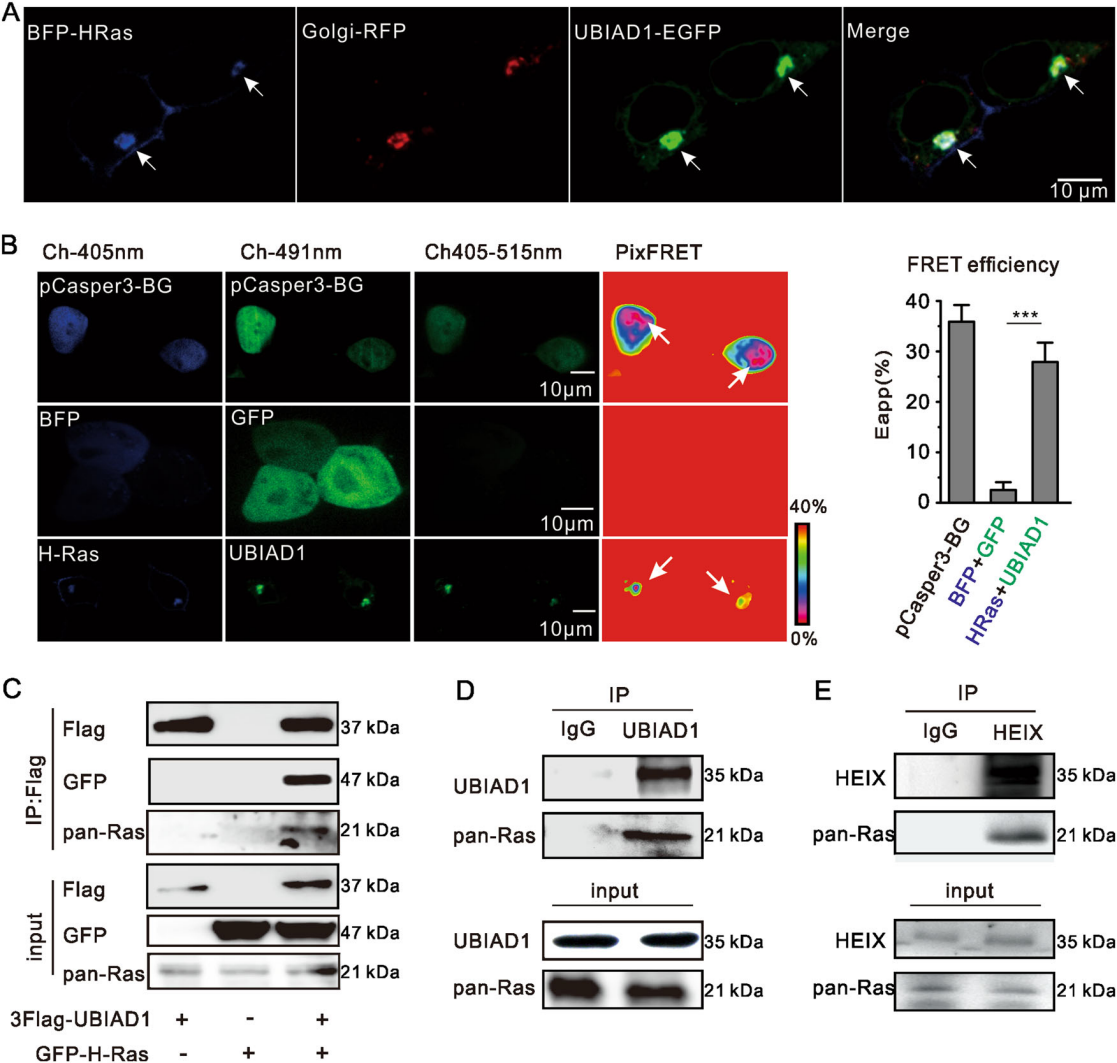
Physical interaction between UBIAD1 and H-Ras. **a** Ectopically expressed UBIAD1 colocalized with H-Ras in the Golgi apparatus. HEK293T cells were transfected with UBIAD1-GFP, Golgi-RFP and BFP-H-Ras, followed by 24 h of cell culture and confocal analysis. The same experiment was repeated three times. **b** Fluorescence resonance energy transfer (FRET) between UBIAD1 and H-Ras. HEK293T cells were transfected with plasmids as indicated, followed by 24 h of culture, confocal microscopy and PixFRET analysis by ImageJ software. The arrows indicate the FRET signal. Positive control: pCasper-BG (a standard probe showing the BFP-GFP FRET phenomenon). Negative control: BFP and GFP. ***p < 0.001, Student’s *t*-test, (*n* = 3 experiments, each with 50 cells). **c** Interaction of UBIAD1 with H-Ras by co-IP. HEK293T cells were transiently transfected with plasmids as indicated. Forty-eight hours after transfection, the cell lysate was exposed to antibodies and analyzed by IP and IB as indicated. The same experiment was repeated three times. **d** Interaction of endogenous UBIAD1 with pan-Ras by co-IP. The total cell lysate from HEK293T cells was exposed to rabbit anti-UBIAD1 antibody and analyzed by IP, followed by IB. Rabbit IgG was used as a negative control. The same experiment was repeated three times. **e** Interaction of endogenous HEIX with pan-Ras in wild-type *Drosophila* larvae by co-IP. The total cell lysate from larvae was exposed to rabbit anti-HEIX antibody or rabbit IgG and analyzed by IP followed by IB as indicated. The same experiment was repeated three times

Considering that UBIAD1 directly acts on Ras signaling and trafficking, we investigated the possibility that UBIAD1 interacts with H-Ras in the Golgi apparatus. When UBIAD1 and H-Ras were overexpressed in HEK293T cells, both molecules colocalized in the Golgi apparatus (Fig. 4a). Consistently, a small portion of the staining pattern of endogenous UBIAD1 overlapped with endogenous Ras in HEK293T cells (Supplementary Fig. S3a).

To provide evidence that UBIAD1 forms a complex with H-Ras, a fluorescence resonance energy transfer (FRET) assay was employed to detect this molecular interaction. Significant FRET signals were detected with 405-515 microscope channels when HEK293T cells were cotransfected with UBIAD1 and H-Ras (Fig. 4b). To determine the possibility of false positives induced by high expression of the protein, we measured FRET signals of UBIAD1/apoE and UBIAD1/Golgi-marker as controls (Supplementary Fig. S3b). FRET between UBIAD1 and apoE, which have confirmed interactions^38^, served as the positive control, and FRET between UBIAD1-GFP and Golgi-BFP was the negative control. To further confirm interaction between UBIAD1 and H-Ras, bimolecular fluorescence complementation (BIFC) and coimmunoprecipitation (co-IP) assays were performed. BIFC signals were detected only in HEK293T cells cotransfected with UBIAD1 and H-Ras (Supplementary Fig. S3c). In addition, immunoprecipitation of UBIAD1 could pull-down H-Ras (Fig. 4c), and vice versa (Supplementary Fig. S3d, e). Ectopically expressed UBIAD1 interacted with Ras but not with Rab11B (a small GTP-binding protein in the Golgi apparatus) in T24 cells (Supplementary Fig. S4a). UBIAD1 strongly interacted with H-Ras^G12V^ compared to its interaction with H-Ras (Supplementary Fig. S4b, c). This finding can explain the more significant effects of UBIAD1 on H-Ras^G12V^ than on H-Ras in inhibiting of the Ras/ERK signaling (Fig. 1g). Furthermore, endogenous Ras and UBIAD1 interacted with each other in HEK293T cells (Fig. 4d). A small portion of HEIX was located in the Golgi apparatus of the blood vasculature (Supplementary Fig. S5), and endogenous HEIX interacted with Ras in wild-type *Drosophila* larvae (Fig. 4e). These data show that UBIAD1 interacts with H-Ras.

### UBIAD1 interacted with the C-terminus of H-Ras

**Fig. 5:**
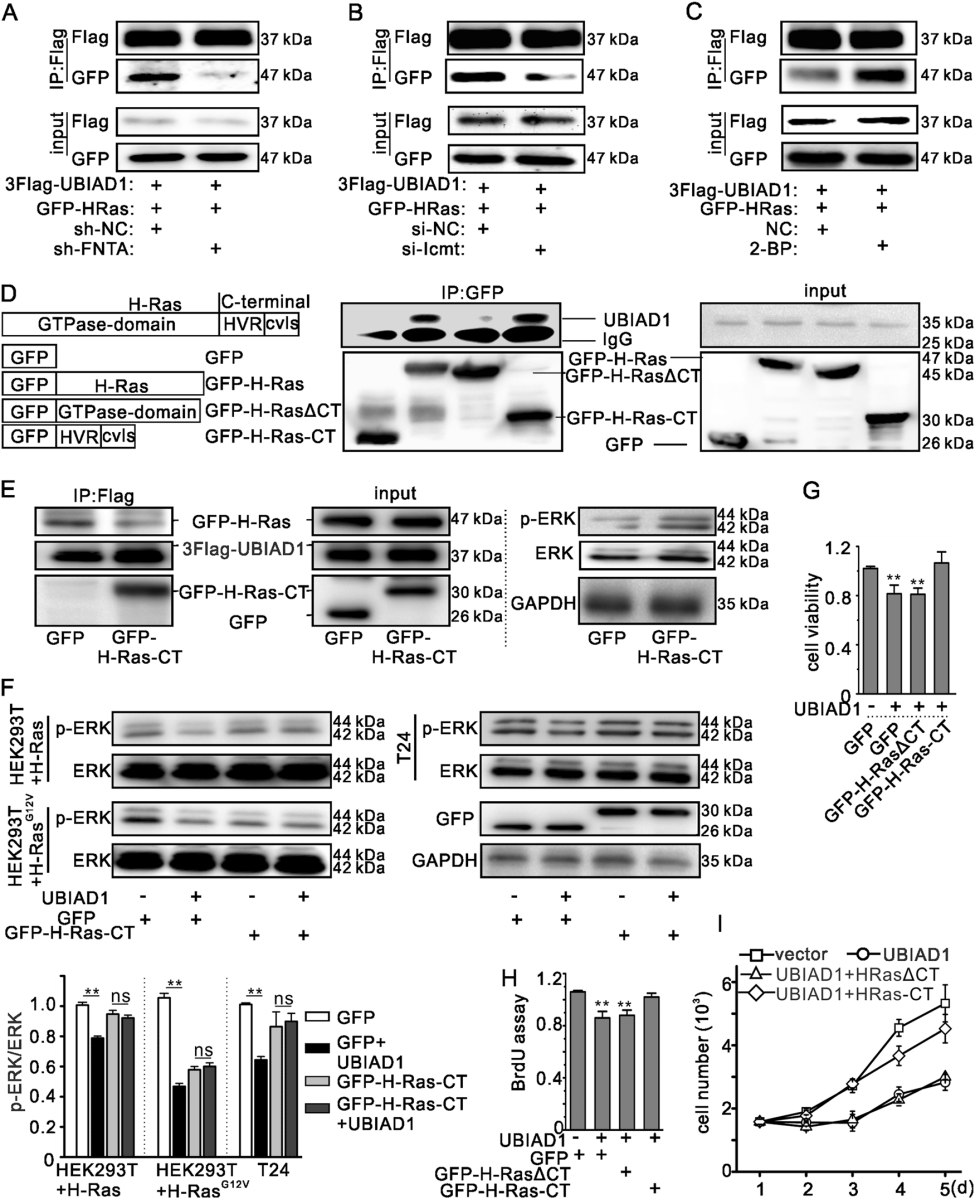
UBIAD1 interacted with the C-terminus of H-Ras. **a** FNTA knockdown decreased interaction between UBIAD1 and H-Ras. HEK293T cells were transfected with 3Flag-UBIAD1 and GFP-H-Ras with or without sh-FNTA. Seventy-two hours after transfection, the cell lysate was exposed to antibodies and analyzed by IP and IB as indicated. The same experiment was repeated three times. **b** Icmt knockdown decreased interaction between UBIAD1 and H-Ras. HEK293T cells were transfected with 3Flag-UBIAD1 and GFP-H-Ras with or without si-Icmt. Seventy-two hours after transfection, the cell lysate was exposed to antibodies and analyzed by IP and IB as indicated. The same experiment was repeated three times. **c** 2-BP treatment increased interaction between UBIAD1 and H-Ras. HEK293T cells were transfected with 3Flag-UBIAD1 and GFP-H-Ras, followed by 72 h of culture and 24 h of culturing with or without 2BP. The cell lysate was exposed to antibodies and analyzed by IP and IB as indicated. The same experiment was repeated three times. **d** Interaction of UBIAD1 with truncated H-Ras. HEK293T cells were transiently transfected with plasmids as indicated. Forty-eight hours after transfection, the cell lysate was exposed to antibodies and analyzed by IP and IB as indicated. The same experiment was repeated three times. **e** The C-terminus of H-Ras decreased interaction between UBIAD1 and H-Ras. HEK293T cells were transiently transfected with plasmids as indicated. Forty-eight hours after transfection, the cell lysate was exposed to antibodies and analyzed by IP and IB as indicated. The same experiment was repeated three times. **f** The C-terminus of H-Ras blocked UBIAD1 function with decreases in p-ERK levels. HEK293T or T24 cells were transfected with plasmids as indicated. Twenty-four hours after transfection, the total cell lysate was analyzed by WB. The lower panel presents the ratio (mean ± SD) from densitometry analyses. **p < 0.01, ns: not significant, Student’s *t*-test, *n* = 3 experiments. **g** The C-terminus of H-Ras suppressed the UBIAD1-induced decrease in T24 cell viability. T24 cells were transfected with plasmids as indicated. Cell viability was detected by the MTT assay. **p < 0.01, Student’s *t*-test, *n* = 3 experiments. **h** The C-terminus of H-Ras inhibited the UBIAD1-induced decrease in T24 cell proliferation. T24 cells were transfected with the plasmids as indicated. Cell proliferation was detected using the BrdU cell proliferation ELISA kit. **p < 0.01, Student’s *t*-test, *n* = 3 experiments. **i** The C-terminus of H-Ras inhibited the UBIAD1-induced decrease in T24 cell growth. T24 cells were transfected with plasmids as indicated. Cell growth was detected by cell counting, *n* = 3 experiments

In normal cells, H-Ras is farnesylated by FTase in the cytoplasm. Then, the VLS of H-Ras is removed by Ras-converting enzyme (Rce1), and the terminal prenylated cysteine residue is methylated by isoprenylcysteine methyltransferase (Icmt) in the ER. Finally, the complex is palmitoylated by palmitoyltransferase in the Golgi apparatus and transported to the plasma membrane^46^. To assess the effect of H-Ras trafficking on the interaction between UBIAD1 and H-Ras, we performed knockdown and inhibition assays. We found that interaction between UBIAD1 and Ras decreased following knock down of FTNA (α-domain of FTase and GGTase) (Fig. 5a) and Icmt (Fig. 5b), respectively, but increased with 2-BP (the inhibitor of palmitoyltransferase) (Fig. 5c), indicating that UBIAD1 interacts with H-Ras in the Golgi apparatus.

To determine the binding region of H-Ras for UBIAD1, a truncated H-Ras protein was fused with GFP. As shown in Fig. 5d and Supplementary Fig. S4d, the C-terminus of H-Ras interacted with UBIAD1. To confirm the contribution of the UBIAD1/H-Ras complex to Ras signaling, the C-terminus of H-Ras (GFP-HRas-CT) was used as a competitive inhibitor to block interaction between UBIAD1 and H-Ras. GFP-HRas-CT decreased interaction between UBIAD1 and H-Ras, and increased the level of p-ERK (Fig. 5e). In addition, UBIAD1 could not decrease the H-Ras-induced or H-Ras^G12V^-induced p-ERK in cells overexpressing H-Ras-CT (Fig. 5f). GFP-H-Ras-CT blocked the inhibition of UBIAD1 in T24 cell viability (Fig. 5g) and proliferation (Fig. 5h, i). Hence, UBIAD1/HEIX physically interacts with Ras, and this interaction inhibits Ras signaling and T24 cell proliferation.

### Geranylgeranyl pyrophosphate (GGPP) was required for interaction between UBIAD1 and H-Ras in the Golgi apparatus

**Fig. 6:**
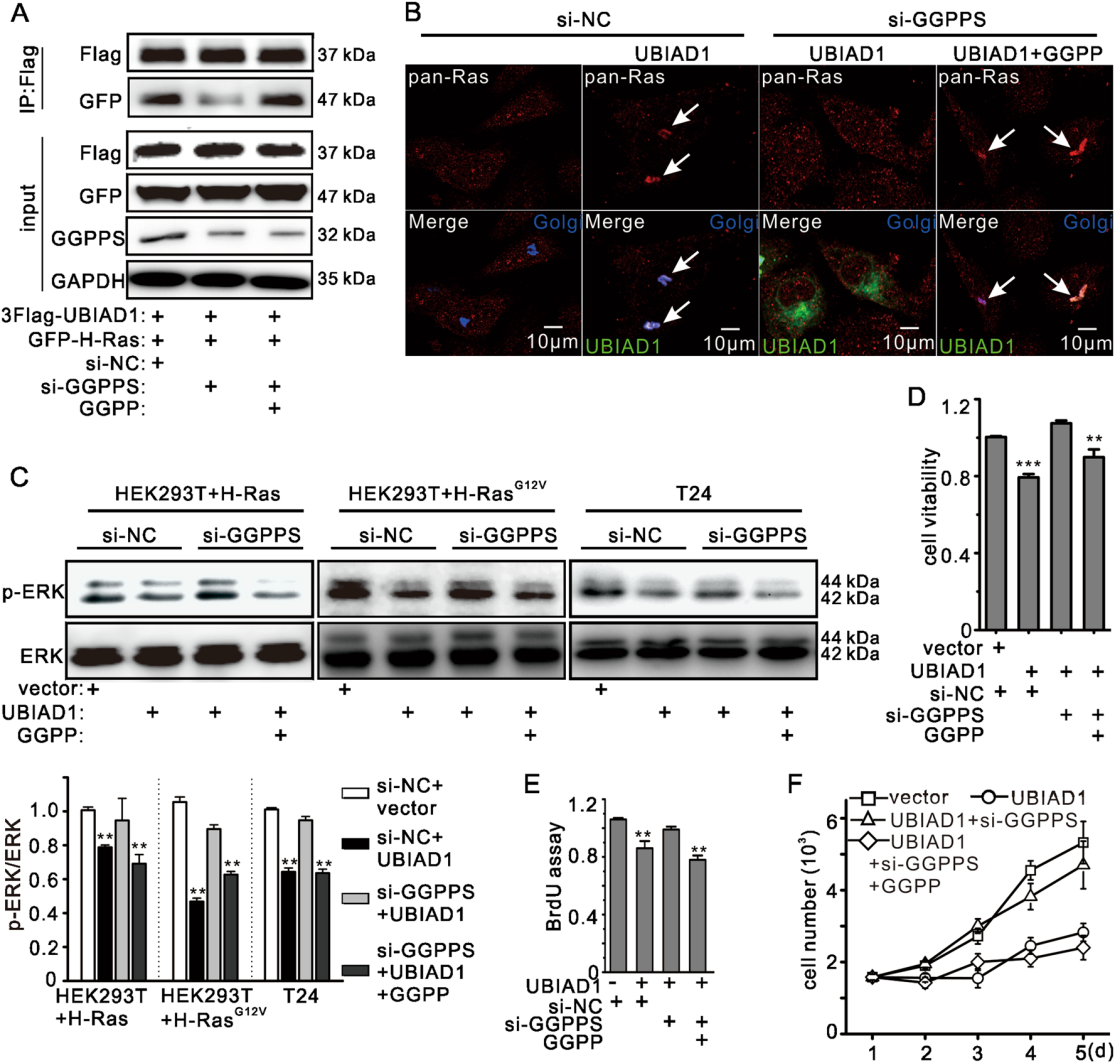
GGPP is essential for the function of UBIAD1 in the Ras/ERK signaling pathway. **a** GGPP supplementation prevented a reduction in interaction between UBIAD1 and H-Ras in the absence of GGPPS. HEK293T cells were transfected with 3Flag-UBIAD1, GFP-H-Ras, and si-GGPPS as indicated, followed by 72 h of culturing with or without GGPP, and all lysates were analyzed by IP and IB. The same experiment was repeated three times. **b** GGPPS knockdown abolished UBIAD1-induced pan-Ras retention in the Golgi. T24 cells were transfected with si-GGPPS and after 48 h, the same cells were transfected with the UBIAD1-GFP construct. The cells were cultured with or without GGPP, followed by staining for pan-Ras and confocal analysis. The same experiment was repeated three times. **c** GGPPS knockdown abolished the UBIAD1-induced decrease in ERK phosphorylation. HEK293T or T24 cells were transfected with si-GGPPS and after 48 h, the same cells were transfected with plasmids as indicated. The cells were cultured with or without GGPP. The total cell lysate was analyzed by WB. The lower panel presents the ratio (mean ± SD) from densitometry analyses. **p < 0.01, Student’s *t*-test, *n* = 3 experiments. **d** GGPPS knockdown suppressed the UBIAD1-induced decrease in T24 cell viability. T24 cells were transfected with si-GGPPS and after 48 h, the same cells were transfected with pcDNA3.1-UBIAD1. The cells were cultured with or without GGPP. Cell viability was detected by the MTT assay. **p < 0.01, ***p < 0.001, Student’s *t*-test, *n* = 3 experiments. **e** GGPPS knockdown suppresses the UBIAD1-induced decrease in T24 cell viability. T24 cells were transfected with si-GGPPS and after 48 h, the same cells were transfected with pcDNA3.1-UBIAD1. The cells were cultured with or without GGPP. Cell proliferation was detected using the BrdU cell proliferation ELISA kit. **p < 0.01, Student’s *t*-test, *n* = 3 experiments. **f** GGPPS knockdown suppresses the UBIAD1-induced decrease in T24 cell growth. T24 cells were transfected with si-GGPPS and after 48 h, the same cells were transfected with pcDNA3.1-UBIAD1. The cells were cultured with or without GGPP. Cell growth was detected by cell counting, *n* = 3 experiments

UBIAD1 utilizes GGPP as a source of geranylgeranyl side-chains during biosynthesis of MK-4^22^ and CoQ10^27^. Statins (simvastatin) can inhibit the mevalonate pathway via 3-hydroxy-3-methylglutaryl-coenzyme A reductase, leading to a simultaneous decrease in the production of FPP (farnesyl pyrophosphate) and GGPP^27,33,47,48^. In our study, we found that statins abrogated the influence of UBIAD1 on the Ras/ERK signaling pathway (Supplementary Fig. S6a, b). However, statins were also able to disrupt the function of Ras because FPP is necessary for the post-translational modification of Ras (Supplementary Fig. S6c). Therefore, statins can block the function of both UBIAD1 and Ras, consistent with those of previous studies^40,47^. This event is prevented by knocking down geranylgeranyl diphosphate synthase (GGPPS), which synthesizes GGPP from FPP^49^. Thus, knockdown of GGPPS decreased interaction between UBIAD1 and H-Ras, which was abrogated by supplementary GGPP (Fig. 6a). Moreover, a lack of GGPPS abolished UBIAD1-induced H-Ras retention in the Golgi apparatus (Fig. 6b and Supplementary Fig. S7). To further confirm whether GGPP was required for the proper function of UBIAD1 on Ras/ERK signaling and cell proliferation, p-ERK, cell viability and cell proliferation were evaluated under a lack of GGPPS. As shown in Fig. 6c–f, GGPPS knockdown attenuated the decrease in p-ERK level and abrogated the inhibition of cell viability and cell proliferation after UBIAD1 transfection for 24 h. This result suggests that GGPPS knockdown interfered with the ability of UBIAD1 to regulate Ras/ERK signaling. Moreover, supplementary GGPP rescued the effect of GGPPS knockdown (Fig. 6a–f and Supplementary Fig. S7), indicating that GGPP contributes to the function of UBIAD1 in the Ras/ERK signaling pathway.

### Mutations of UBIAD1 induced a loss of UBIAD1 function in Ras/MAPK signaling in the Golgi apparatus

**Fig. 7:**
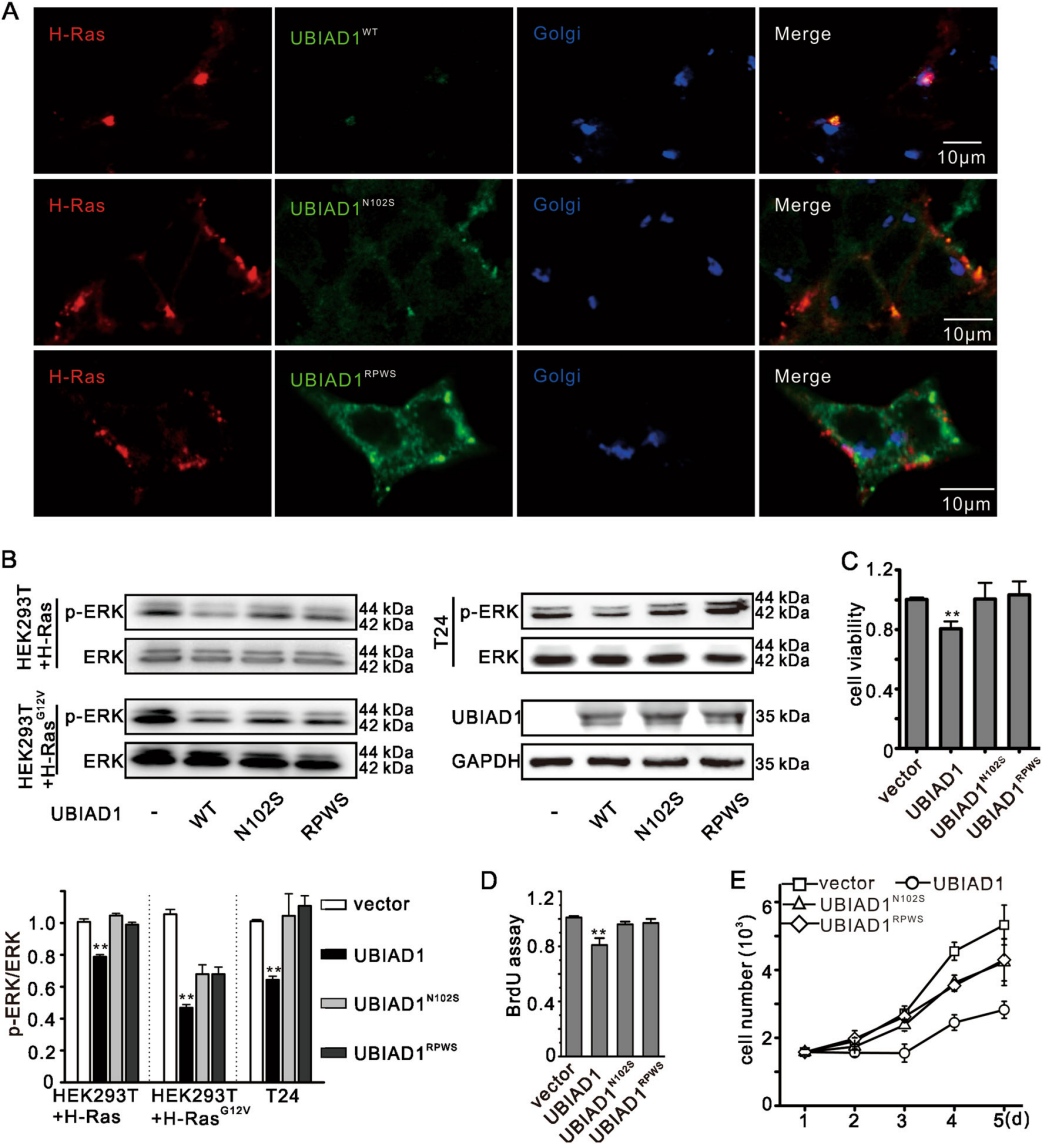
UBIAD1 mutation led to loss of function in Ras/ERK signaling. **a** A mutation in UBIAD1 (N102S, RPWS > AAAA) could not induce H-Ras retention in the Golgi. UBAD1^N102S^ cannot use GGPP as a substrate, and UBIAD1^RPWS^ is not located in the Golgi. HEK293T cells were transfected with plasmids as indicated and after 24 h of transfection, confocal analysis was performed, the same experiment was repeated three times. **b** A UBIAD1 mutation did not decrease phosphorylated ERK. Cells were transfected with plasmids and 24 h after transfection, total cell lysate was analyzed by WB. The same experiment was repeated three times. **c** A UBIAD1 mutation did not decrease T24 cell viability. T24 cells were transfected with plasmids and 24 h after transfection, cell viability was detected by the MTT assay. ***p < 0.001, Student’s *t*-test, *n* = 3 experiments. **d** A UBIAD1 mutation did not decrease T24 cell proliferation. T24 cells were transfected with plasmids and 24 h after transfection, cell proliferation was detected by BrdU cell proliferation ELISA kit. ***p < 0.001, Student’s *t*-test, *n* = 3 experiments. **e** A UBIAD1 mutation did not decrease T24 cell growth. T24 cells were transfected with plasmids and 24 h after transfection, cell growth was detected by cell counting, *n* = 3 experiments

Our study showed that UBIAD1 induced H-Ras retention in the Golgi apparatus via GGPP. UBIAD1 mutants were then used to confirm this observation. UBIAD1^N102S^ is not able to bind to GGPP and be transported to the Golgi apparatus^47,50^, and UBIAD1^RPWS^ does not localize in the Golgi apparatus^28^. Figure 7a shows that UBIAD1 mutants did not localize in the Golgi apparatus, which is consistent with previous studies^28,33^. Interestingly, H-Ras was located in the Golgi apparatus in the presence of UBIAD1^WT^, while it was located in the plasma membrane in the presence of UBIAD1^N102S^ and UBIAD1^RPWS^. UBIAD1 mutation did not affect ERK phosphorylation (Fig. 7b) and inhibit T24 cell viability (Fig. 7c). In addition, mutations of UBIAD1 did not influence T24 cell proliferation (Fig. 7d, e), indicating that UBIAD1 mutation trigger its loss of function in regulating the Ras/ERK signaling pathway. These findings demonstrate that UBIAD1 inhibits H-Ras in the Golgi apparatus via GGPP.

## Discussion

In this study, it is reported that UBIAD1 interacts with the C-terminus of H-Ras, regulates the plasma membrane-Golgi cycle of H-Ras, and limits activation of H-Ras signaling in the plasma membrane which consequently inhibits cell growth.

Ras mutations are found in approximately 25% of human tumors^51^, and Ras oncogenic activity is dependent on the protein’s association with the inner face of the plasma membrane. Therefore, preventing Ras from trafficking to the plasma membrane is an important direction for anti-cancer and anti-Ras drug discovery^52^.

Farnesyl pyrophosphate is added to the CAAX cysteine of Ras by farnesyltransferase, and the AAX amino acids of the CAAX motif are substrates for an endoprotease designated Rce1. Following Rce1-mediated proteolysis, the C-terminal prenylcysteine of Ras is methylesterified by Icmt^53^. Due to the different HVR domains, H-Ras, N-Ras and K-Ras4A are transported to the Golgi apparatus, whereas K-Ras4B is transported to the plasma membrane after being phosphorylated by PKC^54^. H-Ras, N-Ras and K-Ras4A are palmitoylated by palmitoyl acyltransferases on the surface of the Golgi apparatus and then are transported to the plasma membrane^55^. The G-P peptidyl-prolyl bond of H-Ras at position 178–179 undergoes cis-trans isomerization catalyzed by FKBP12 and then HRas is transported back to the Golgi apparatus^15^, representing plasma membrane-Golgi cycling of H-Ras.

FTIs have been developed based on the essential role of the farnesyl lipid modification for all subsequent post-translational modifications and for RAS oncogenic activity^53^. Although FTIs inhibit H-Ras-driven growth of cancer cells, they have no effect on cancer driven by N-Ras or K-Ras because NRas and KRas are modified with the related geranylgeranyl isoprenoid in the presence of FTIs^56^. Inhibition of palmitoylation or increasing the activation of FKBP12 can limit activation of Ras in the plasma membrane, leading to the suppression of cell growth^52^.

One identified target for anti-Ras drugs is the prenyl-binding protein phosphodiesterase δ (PDEδ), which binds to the C-terminus of Ras and facilitates the transit of Ras proteins to either the Golgi apparatus or the recycling endosomes^57^. PDEδ inhibitor treatment shifts Ras from the plasma membrane to the endomembranes, inhibits Ras activation in the plasma membrane and suppresses the growth of cancer cells^58^. In principle, targeting PDEδ would overcome the concerns encountered with FTIs.

Here, we found that UBIAD1 interacted with the C-terminus of H-Ras, which is consistent with the binding data for FKBP12 and PDEδ^15,58^. UBIAD1 inhibited H-Ras trafficking from the Golgi apparatus to the plasma membrane and inhibited the proliferation of bladder cancer cells. Ras translocates to the plasma membrane in the absence of UBIAD1, leading to the formation of cancer. Therefore, UBIAD1 has a similar function as PDEδ inhibitors or FKBP12 in regulating Ras trafficking between the plasma membrane and the Golgi apparatus (or ER). UBIAD1 may be a potential target for regulating Ras trafficking.

Ras is an important protein implicated in cell proliferation circuits, and can influence other cell circuits, such as motility, cytostasis, differentiation, and viability circuits^4,5^. Ras sends different signal outputs in various regions such as the plasma membrane, Golgi apparatus, ER and mitochondria^59^. These events may occur through several factors, such as the different MAPK scaffolds employed by the plasma membrane and the Golgi apparatus^3^. Ras/ERK signaling activated by Ras in the plasma membrane induces cell growth, and Ras in the Golgi can regulate the Raf/ERK, PI3K/AKT/mTOR or PLC-γ-Ca^2+^ signaling pathway, leading to cell differentiation^59,60^. PAQR9/10 bound to H-Ras in the Golgi retains H-Ras in the Golgi and induces cell differentiation^9^. ENOS activates N-Ras in the Golgi and induces T-cell dependent apoptosis^61^. Additionally, FKBP12 promotes H-Ras trafficking from the plasma membrane to the Golgi, activates H-Ras in the Golgi, and induces cell differentiation under EGF treatment^15^. Our findings show that UBIAD1 prevents H-Ras trafficking from the Golgi to the plasma membrane. UBIAD1 is essential in embryonic mouse development^30^, and *heix* is specifically expressed in a small cluster of hemocytes within the *Drosophila* embryonic head mesoderm, a developmental zone of hemocytes^19^. These findings suggest a new function of the UBIAD1 protein in cell differentiation during biological development. UBIAD1 may regulate the balance between cell proliferation and differentiation by mediating plasma membrane- Golgi cycling of H-Ras.

UBIAD1 suppresses tumor growth in bladder cancer^20^, prostate cancer^21^, and renal carcinoma^31^. UBIAD1 can inhibit the cell cycle and cell proliferation by regulating cholesterol and SXR target genes in the ER or mitochondria^26,62^. Here, the results demonstrate that UBIAD1 inhibits cell growth by regulating Ras/ERK signaling in the Golgi apparatus, suggesting that UBIAD1 is involved in the inhibition of cell proliferation. UBIAD1 is a membrane-associated protein with different functions in the ER and Golgi apparatus^27,33,47^, and GGPP is necessary during the transport of this protein from the ER to the Golgi apparatus^63^. When GGPP is insufficient, the transport of UBIAD1 to the ER under treatment with statins or GGPPS knockdown induces HMG-CoA reductase synthesis of GGPP and cholesterol, causing cell proliferation^33^. By contrast, transport of UBIAD1 to the Golgi apparatus in the presence of GGPP causes the protein to bind to H-Ras, which consequently prevents the trafficking of H-Ras from the Golgi apparatus to the plasma membrane and inhibits cell growth. UBIAD1 mutants lack the ability to use GGPP and are therefore located in the ER instead of the Golgi apparatus, inducing cholesterol generation and SCCD disease^33^. These data show that UBIAD1 mutation or wild-type UBIAD1 without GGPP are localized in the ER and both types lose their functionality, consistent with previous studies. Therefore, UBIAD1 is in a dynamic balance between the ER and the Golgi apparatus to regulate cell growth.

The establishment of carcinoma is a complex process. Ectopic expression of H-Ras can induce melanoma in mice^18^, and a lack of *heix* can also cause melanotic mass formation in *Drosophila* larvae^19^. Therefore, H-Ras and UBIAD1 may be associated with melanoma formation. Our data show that large amounts of H-Ras aggregate in the plasma membrane and activate Ras/ERK signaling, causing melanotic mass formation under *heix*-deficient conditions. The mechanism responsible for melanotic mass formation in *Drosophila* may be similar to that in mice. Melanotic masses, the most obvious feature of *heix* mutation in *Drosophila*^19^, can be subdivided into melanotic nodules engaging the hemocyte-mediated encapsulation and melanizations that are not encapsulated by hemocytes^64^. Loss of function of heix or gain of function of Ras leads to an increased proliferation of hemocytes^19,45^. Hemocyte proliferation is closely correlated with the formation of melanotic mass, which can be attributed to an increase in the number of crystal cells associated with melanization^65,66^. Our study show that *heix* mutant leads to activation of Ras signaling on the plasma membrane. Therefore, the formation of melanotic mass in a *heix* mutant may result from Ras-induced proliferation of hemocytes. Activated Ras reportedly increases macropinocytosis and autophagy and directs glucose metabolism into hexosamine biosynthetic pathways by upregulating many key enzymes involved in glycolysis^51^. Therefore, UBIAD1/HEIX knockdown induces Ras/MAPK signaling and might reprogram metabolism, leading to tumorigenesis. In addition, it is reported that the melanotic mass formation in *Drosophila* is linked to a hemocyte-mediated immune response^64^. Previous studies report that loss of *heix* function not only activates the Ras signaling pathway but also leads to the activation of immune-related pathways (Toll, JAK/STAT, IMD pathways)^19,66^. Therefore, besides the Ras pathway, the formation of melanotic mass in *heix* mutants may be influenced by immune-related pathways.

In summary, UBIAD1 bind to H-Ras, regulates the H-Ras intracellular trafficking cycle, inhibits Ras/MAPK signaling, suppreses bladder cancer cell proliferation and plays important roles in melanotic mass formation in *Drosophila*. Our findings confirm the tumor suppressor function of UBIAD1 in cellular circuits in melanotic mass formation in *Drosophila*.

## Methods

### Cell culture and cell transfection

Human bladder cancer cells T24 and human embryonic kidney cells HEK293T were obtained from American Type Culture Collection. HEK293T cells were cultured in Dulbecco’s modified Eagle’s medium (Hyclone) supplemented with 10% fetal bovine serum (FBS).T24 cells were cultured in minimum essential medium (MEM, Hyclone) supplemented with 10% FBS. All cultures were maintained at 37 °C in a humidified incubator with 5% CO_2_. Transient transfection of HEK293T and T24 cells was performed with Lipofectamine^TM^ 2000^28^.

### Real-time quantitative PCR analysis

Total RNA was extracted from HEK293T cells with TRIzol reagent (Invitrogen, USA), and cDNA was synthesized using the first-strand cDNA synthesis kit (Eastep RT Master Mix kit Promega, USA). Real-time quantitative PCR was performed using the double-stranded DNA dye SYBR Green (Roche, Switzerland) to quantify the amount of gene expression. Gene expression assays were performed using procedures recommended by ABI Biosciences, and data were analyzed using ΔΔCt values. Primer pairs for UBIAD1 were used according to a previous study^67^

(UBIAD1-F:5'-GTGTGCCTCCTACGTGTTGGCC-3'; UBIAD1-R:5'-AAATTACCGGCCCCGTGCACAG-3').

The mRNAs levels were normalized to ACTIN.

The primer pairs of ACTIN:

(ACTIN-F:5'-CGCGAGAAGATGACCCAGAT-3'

ACTIN-R:5'-GTACGGCCAGAGGCGTACAG-3').

### Plasmid constructs

The plasmid pOTB-UBIAD1 was purchased from Open Biosystems Inc. Enhanced green fluorescent protein (EGFP) vectors pEGFP-N1, pEGFP-C1, pCasper3-BG (TAGBFP-GFP), mammalian expression vector pcDNA3.1 and TAGBFP were obtained from Invitrogen. pDsRed-Golgi vector was obtained from Clontech, previously described^28^. For construction of DsRed-H-Ras, full-length human H-Ras cDNA was amplified by PCR and cloned with DsRed-Monomer into the pcDNA3.1 vector. BFP-H-Ras was constructed by fusing H-Ras at the N-terminus of BFP into the pcDNA3.1 vector. GFP-H-Ras and its mutation were cloned into pEGFP-C1. Flag-H-Ras and its mutation were constructed by fusing the Flag tag to the N-terminus of H-Ras and inserting into pcDNA3.1. The plasmid 3Flag-UBIAD1 was constructed by fusing the 3Flag tag to the N-terminus of UBIAD1 and inserting into pcDNA3.1. pGFP-RBD was kindly provided by Dr. Mark R. Philips. Full-length human UBIAD1 and Apo E cDNA fragment were separately cloned into the pEGFP-N1 vector to create UBIAD1-EGFP and Apo E-EGFP. UBIAD1 was cloned into pcDNA3.1 to generate pcDNA3.1-UBIAD1. UBIAD1-BFP, Golgi-BFP and Apo E-BFP were constructed by fusing BFP with the C-terminus of UBIAD1 and Apo E and inserting into pcDNA3.1. All vectors for the BIFC assay were constructed using pcDNA3.1.

### Immunofluorescence (IF) and confocal microscopy

HEK293T cells were mounted onto the polylysine-slides and were 4% formaldehyde fixed (10 min) to permeabilize cells. Cells were incubated in 1% BSA (Bovine Serum Albumin)/10% normal goat serum in 0.1% PBS (Phosphate Buffer Saline)-Tween for 2 h to block non-specific protein-protein interactions. Cells were then incubated with primary antibodies overnight at 4 °C. After several washes with cold PBS, the slides were incubated with secondary antibodies for 2 h at room temperature. Fluorescence was observed by confocal microscopy (FV1000, Olympus) using 488 argon ion laser for GFP/488, a 543 He-Ne laser for RAF/549, and a 405 diode laser for BFP/DAPI. FLUOVIEW (Olympus) was used as the image acquisition software. Images were acquired, processed and analyzed with Image J (National Institutes of Health).

### EGF treatment and inhibitor assay

Cells were starved for 6 h, cultured with DMEM and stimulated with 100 ng/ml EGF for different periods of time. The inhibitors U0126 (Medchem Express, USA, #HY12031), FTI-277 (Sigma, USA, #F9803), Salirasib (Medchem Express, USA, #HY14754), 2BP (Sigma, USA, #238422), tunicamycin (Medchem Express, USA, #HY13585), and simvastatin (Cayman, USA, #10010344) were dissolved in DMSO (Dimethyl sulfoxide). The working concentrations for cells and *Drosophila* were as follows: U0126 (10 μM), FTI-277 (10 μM), Salirasib (35 μM), 2BP (150 μM), tunicamycin (10 μM), and simvastatin (10 μM). FPP (#63250) and GGPP (#63330) were purchased from Cayman.

### Antibodies

The following antibodies were used in this study: p-c-Raf (Ser259) (#9421), p-c-Raf (Ser338) (#9427), p-c-Raf (Ser289/296/301) (#9431), c-Raf (#9422), p-ERK1/2 (#4370), total-ERK1/2 (#4695), and caspase-3 (#9662) from Cell Signaling Technology (USA); anti-phospho-MEK (Ser218/222)/MEK2 (Ser222/226) (#2283379) from Millipore (USA); MEK1/2 (Ab-217/221) (#21203) antibody from Signalway Antibody (USA); Antibodies detecting actin (A01010-1) and GAPDH (A01020-1) from Abbkine (USA); Flag tag monoclonal antibody (A00187) from GenScript (China); GFP tag antibody (#66002-1) and HMGB1 (#10829-1) from proteintech (China); 488/549/645-conjugated secondary antibody from Abbkine (USA); HRP-conjugated secondary antibody from ABGENT (USA); UBIAD1 antibody (ab36832), Pan-Ras antibody (ab16907), and LC3B (AB192890) from Abcam (USA); rabbit IgG, mouse IgG from Immunoreagents (USA). Antibodies used for experiments with *Drosophila* were as follows: p-ERK (sc-7976) and ERK2 (sc-153) from Santa Cruz (US), pan-Ras (#3339) from Cell Signaling Technology (USA). HEIX antibodies were synthesized by GL Biochem (Shanghai, China), as previously described^19^.

### Immunoprecipitation and immunoblotting

An immunoprecipitation assay was performed 48 h after cells were transfected with plasmids. Cells were lysed in lysis buffer (50 mM Tris-HCl (pH = 7.4), 150 mM NaCl, 0.5 mM EDTA and 0.1% NP-40, supplemented with protease inhibitor cocktail (CST #5871S) and phosphorylated protease inhibitor cocktail (BOSTER #AR119)), frozen and thawed once. Cell lysates and specific antibodies were incubated at 4 °C with continuous mixing overnight. Fifty microliters protein G-coupled beads (Millipore, USA, LSKMAGG02) were prepared (according to the supplier’s instructions), and the mixture containing the pre-formed antibody-antigen complex was added to the beads. After 3 h of incubation at 4 °C, the supernatant was removed, and the beads were washed five times with cold PBS containing 0.1% Tween 20. Following the final wash, 60 μl sample buffer was added and the reaction was heated at 90 °C. The sample was detected by western blotting as previously described by Xia and collaborators^39^. In short, proteins were separated by sodium dodecyl sulphate-polyacrylamide gel electrophoresis (12% gel) and transferred to PVDF (Polyvinylidene fluoride) membrane. The membranes were subsequently blocked with 5% fat-free milk dissolved in TBS (Triethanolamine Buffer Saline) containing 0.1% Tween for 3 h at room temperature and then incubated overnight with primary antibodies at 4 °C. After incubation with horseradish peroxidase-conjugated secondary antibody, proteins were detected using enhanced chemiluminescence (ECL). Band intensity was quantified with Quantity One software.

### RNA interference

shRNA vector-pGPU6/GFP/Neo was constructed by GenePharma (Suzhou, China). sh-UBIAD1:^26^ 5'-CACCGTAAGTGTTGACAATTACCGGTTCAAGAGACCGGTAATTGTCAACACTTACTTTTTTG-3'.

sh-NC: 5'-CACCGTTCTCCGAACGTGTCACGTCAAGAGATTAACGTGACACGTTCGGAGAATTTTTTG-3'.

sh-FNTA:^68^ The shRNA vector into PGH1/RFP/Neo were constructed by GenePharma. 5'-CACCGATCCGGTGCCGCAGAATGATTCAAGAGATCATTCTGCGGCACCGGATTTTTTTG-3’. sh-NC: 5'-CACCGTTCTCCGAACGTGTCACGTTTCAAGAGAACGTGACACGTTCGGAGAATTTTTTTG-3'.

si-RNA oligonucleotides were constructed by GenePharma (Suzhou, China).

si-UBIAD1:^39^ 5'-GUAAUUUGGUCAACACUUATT-3'

si-GGPPS:^49^ 5'-GUCCCACUGAAGAAGAAUA-3'.

si-Icmt:^69^ 5'-CCAUAGCUUAUAUUCUCAAdTdT-3'.

si-NC: 5'-UUCUCCGAACGUGUCACGUTT-3'.

### Cell viability assay and BrdU cell proliferation ELISA assay

T24 cells were plated in 96-well plates at a density of approximately 10^5^ cells per well; after 24 h, the cells were transfected with plasmids. Cell viability was evaluated using the MTT (CellTiter 96 Aqueous One Solution Cell Proliferation Assay, #G3580, Promega, US) assay, according to the manufacturer’s protocol.

T24 cells were plated in 96-well plates at a density of approximately 10^3^ cells per well; after 24 h, the cells were transfected with plasmids. Cell proliferation was evaluated using the BrdU cell proliferation ELISA kit (colorimetric) (ab126556, Abcam, US), according to the manufacturer’s protocol.

### *Drosophila* stocks and medicine treatment

The stocks used in this study were previously described^19^. *heix*^*k11403*^*/Df* and *heix*^*k11403*^*/heix*^*1*^ are mutations of *heix*. For the P-element allele *heix*^*k11403*^, the insertion site is between bases 2L: 16299582 and 2L: 16299583 (transcription initiation area) and is expected to induce a non-expression (Bloomington stock 11031). For the ethylmethansulfonate (EMS) allele *heix*^*1*^, an AR144 mutation resulting from a G to A base change in the last residue of the only intron and is expected to result in an aberrant splicing event (Bloomington stock 3600). The deficiency allele *Df(2* *L)RA5/CyO* (Bloomington stock 6915). The control is wild-type (*w*^*1118*^), and *heix*^*k11403*^*/Df; tub P* *>* *heix* is the rescue type.

Food for *Drosophila* was prepared in a tube on the first day, and treatments were added to the food surface on the second day. Virgin flies were placed in the tube on the third day, and crossing was performed on the fourth day. The flies were removed from the tube 1 week later.

### Statistical analysis

Results are presented as the mean ± standard deviation (SD). Statistical data comparisons among groups were performed using a non-parametric, Student’s *t*-test, *p* < 0.05 was considered statistically significant. Each experiment was performed at least in triplicate.

## Electronic supplementary material

supplementary figure
