# Supplementary material for: UBIAD1 suppresses the proliferation of bladder carcinoma cells by regulating H-Ras intracellular trafficking via interaction with the C-terminal domain of H-Ras
Source: Cell Death Dis. 2018 Dec 5;9(12):1170. doi: 10.1038/s41419-018-1215-4 (PMC6281600; doi:10.1038/s41419-018-1215-4)
Supplement: Supplementary file 1 — supplementary figure [file 41419_2018_1215_MOESM1_ESM.pdf]

Supplementary Materials for

UBIAD1 suppresses the proliferation of bladder carcinoma cells by regulating H-Ras intracellular trafficking via interaction with the C-terminal domain of H-Ras

Zhiliang Xu[1], Fengsen Duan[1], Huiai Lu[1], Maytham Abdulkadhim Dragh[1], Yanzhi Xia[1], Huageng Liang[2], and Ling Hong[1*]

1 Department of Genetics and Developmental Biology, College of Life Science and Technology, Huazhong University of Science and Technology, Wuhan, Hubei, P. R. China

2 Department of Urology, Union Hospital, Tongji Medical College, Huazhong University of Science and Technology, Wuhan, Hubei, P. R. China

* Corresponding author: Email address: lhong@mail.hust.edu.cn (Ling Hong)

Supplementary figures

Figure-S1

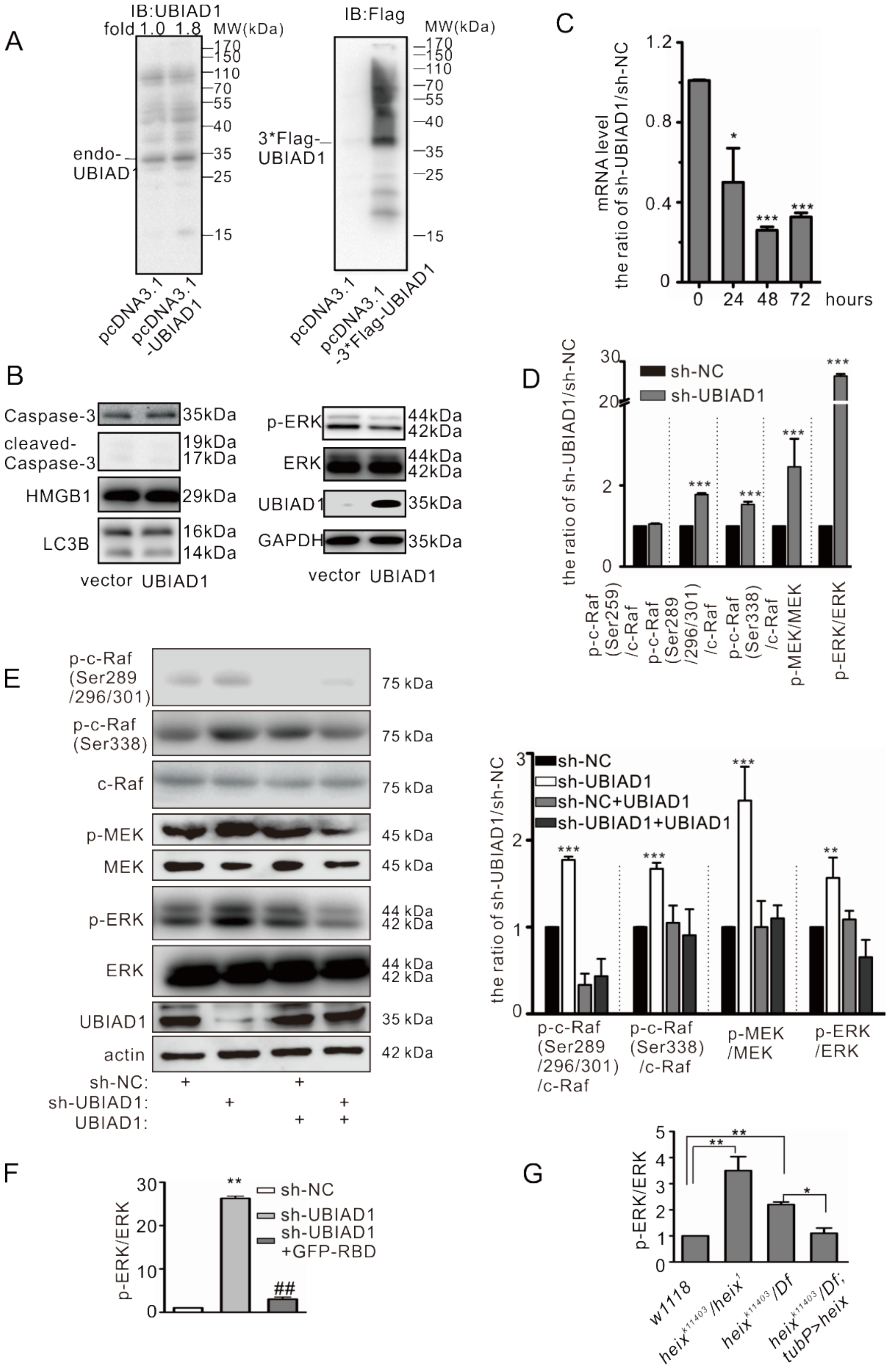

Figure S1 UBIAD1 inhibits Ras/MAPK signalling pathway, Related to Figure 1

(A) The full-western blot of UBIAD1 in HEK293T cells. HEK293T cells were transfected with UBIAD1 or 3*Flag-UBIAD1. After 24 hours, total cell lysate were used by IB. The same experiment was repeated three times.

(B) The marker proteins (apoptosis, necrosis and autophagy) were detected in T24 cells. T24 cells were transfected with increasing amounts of pcDNA3.1-UBIAD1. Twenty-four hours after transfection, total cell lysate was exposed to antibodies and examined by WB. Cleaved caspaes-3 is the maker of apoptosis. HMGB1 is the marker of necrosis. LC3B is the marker of autophagy. The same experiment was repeated three times.

(C) The mRNA level of UBIAD1 in time-course by Q-PCR. HEK293T cells were transfected with sh-UBIAD1 and total RNA was extracted from cells at a different time. *$p<0.05$, ***$p<0.001$, Student's *t*-test, n=3 experiments.

(D) The panel represents ratio (mean ±SD) from densitometer analyses of Figure.1E. ***$p<0.001$, Student's *t*-test, n=3 experiments.

(E) Unregulated Ras/ERK signaling by sh-UBIAD1 is abrogated by UBIAD1 construct that is refractory to RNAi interference. HEK293T cells were transfected with sh-UBIAD1 and after 48 hours the same cells were transfected with increasing amounts of UBIAD1 construct again. Total cell lysate was used in IB with the antibodies as indicated 24 hours later. The right panel represents ratio (mean ±SD) calculated from densitometer analyses of three independent experiments. **$p<0.01$, ***$p<0.001$ compared to the control group which the cells were transiently transfected with the same amounts of sh-NC. Student's *t*-test, n=3 experiments.

(F, G,) The panel represents ratio (mean ±SD) from densitometer analyses of Figure.1F, I. *$p<0.05$, **$p<0.01$, ***$p<0.001$, Student's *t*-test, n=3 experiments.

Figure-S2

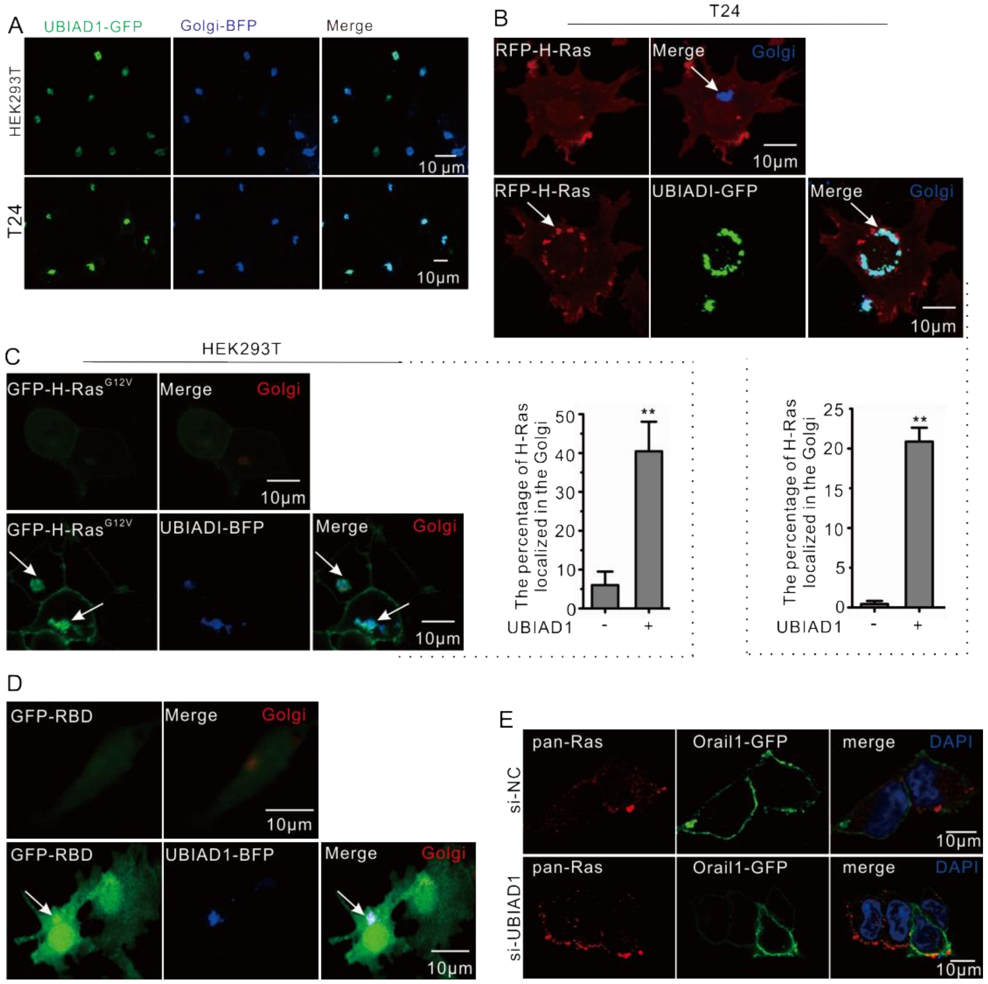

Figure S2 UBIAD1 regulates H-Ras trafficking in the Golgi apparatus, Related to Figure 2

(A) Ectopically expressed UBIAD1 is localized in the Golgi apparatus. HEK293T and T24 cells were transfected with UBIAD1-GFP and Golgi-BFP (Golgi marker), followed by 24 hours of culturing and confocal analysis. The same experiment was repeated three times.

(B) UBIAD1 increased H-Ras retention in Golgi apparatus in T24 cells. T24 cells were transfected with RFP-H-Ras with or without UBIAD1-GFP, followed by 24 hours of culturing and confocal analysis. The lower panel shows the percentage of Ras localized in the Golgi, **p<0.01 as compared to control, (n=3 experiments, each with 100 cells).

(C) UBIAD1 increased H-Ras^{G12V} retention in Golgi apparatus in HEK293T cells. HEK293T cells were transfected with GFP-H-Ras^{G12V}, with or without UBIAD1-BFP, followed by 24 hours of culturing and confocal analysis. The right panel shows the percentage of Ras localized in the Golgi, **p<0.01 as compared to control, (n=3 experiments, each with

100 cells).

(D) UBIAD1 induced endogenous H-Ras^{G12V} retention in Golgi apparatus in T24 cells. T24 cells were transfected with GFP-RBD (a marker of activated Ras) with or without UBIAD1-BFP, followed by 24 hours of culturing and confocal analysis. The same experiment was repeated three times.

(E) Ras is localized in plasma membrane in the deficiency of UBIAD1. HEK293T cells were transfected with si-UBIAD1. Forty-eight hours after transfected, cells were again transfected with Orail 1-GFP (a maker of membrane), followed by 24 hours culturing, staining with pan-Ras antibody, and DAPI and confocal analysis. The same experiment was repeated three times.

Figure-S3

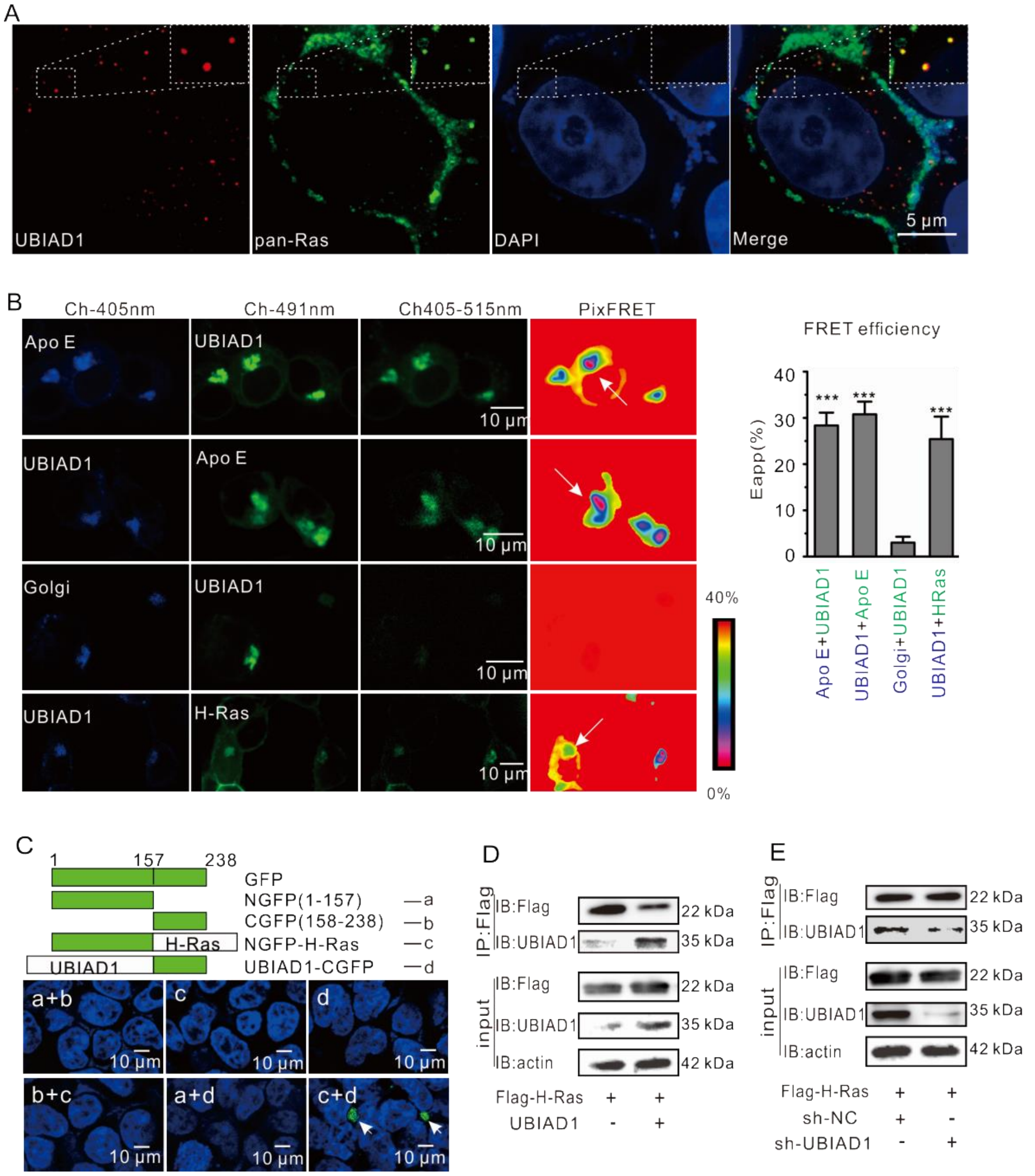

Figure S3 UBIAD1 is localized and interacted with H-Ras, Related to Figure 4

(A) UBIAD1 is localized with H-Ras in HEK293T cells. HEK293T cells were stained with Rabbit-anti-UBIAD1, Mouse-anti-pan-Ras and DAPI. The colocalisation threshold was analyzed by using ImageJ. (m1, m2) of UBIAD1 and Ras is (0.9895, 1.0000). (m1, m2) of UBIAD1 and DAPI is (0.2578, 0.2116). (m1, m2) of Ras and DAPI is (0.5010, 0.4235). n=3 experiments, with each 50 cells.

(B) The FRET of UBIAD1 and H-Ras. HEK293T cells were transfected with plasmids as indicated, followed by 24 hours of culturing, confocal and PixFRET analysis by ImageJ

software. The arrows indicate the FRET signal. Positive control: UBIAD1 and Apo-E, which has been confirmed to interact with each other. Negative control: Golgi-BFP (Golgi-marker) and UBIAD1-GFP. ***$p<0.001$ as compared to negative control, Student's *t*-test, (n=3 experiments, each with 50 cells).

(C) Bimolecular Fluorescence Complementation (BIFC) between UBIAD1 and H-Ras. HEK293T cells were transfected with the plasmids as indicated, followed by 36 hours of culturing in 37°C, twenty-four hours of culturing in 30°C, nuclear staining, and confocal analysis. The arrows indicate the BIFC signal. The same experiment was repeated three times.

(D) Ectopic expression of UBIAD1 increased the interaction between UBIAD1 and H-Ras. HEK293T cells were transfected with Flag-H-Ras, with or without UBIAD1. Forty-eight hours after transfection, total cell lysate was used in IP and IB. The same experiment was repeated three times.

(E) The interaction between UBIAD1 and H-Ras was decreased after knock down of UBIAD1. HEK293T cells were transfected with Flag-H-Ras with or without sh-UBIAD1. Seventy-two hours after transfection, total cell lysate was used in IP and IB. The same experiment was repeated three times.

Figure-S4

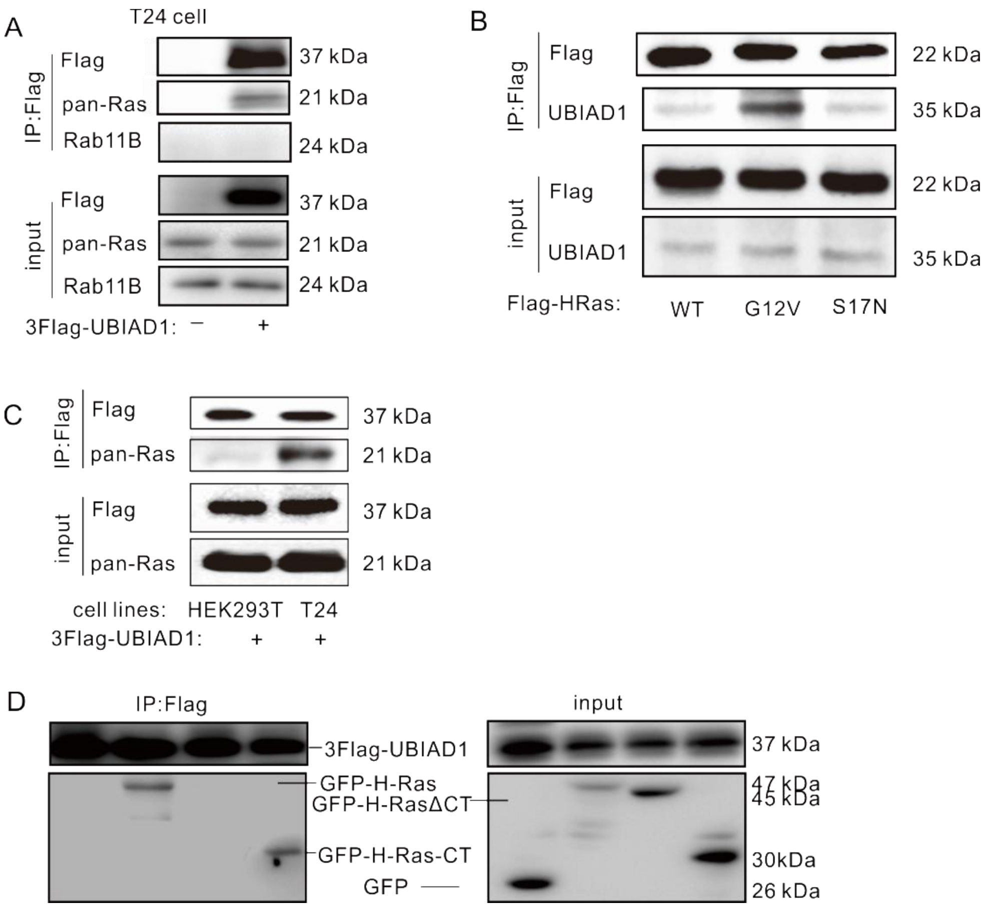

Figure S4 UBIAD1 interacts with H-Ras^{G12V} stronger than H-Ras, Related to Figure 4, 5

(A) UBIAD1 interacted with Ras but without Rab11B. T24 cells were transfected with 3Flag-UBIAD1. Forty-eight hours after transfected, total cell lysate was used in IP and IB. The same experiment was repeated three times.

(B) H-RasG12V interacts with UBIAD1 more easily than H-Ras. HEK293T cells were transfected with plasmids as indicated. Forty-eight hours after transfected, total cell lysate was used in IP and IB. The same experiment was repeated three times.

(C) UBIAD1 tends to combine with the activated H-Ras in T24 cells. HEK293T or T24 cells were transfected with 3Flag-UBIAD1. Forty-eight hours after transfected, total cell lysate was used in IP and IB. The same experiment was repeated three times.

(D) The interaction between UBIAD1 and the C-terminus of H-Ras. HEK293T cells were transiently transfected with the plasmids as indicated. Forty-eight hours after transfection, the cell lysate was exposed to antibodies and analyzed by IP and IB as indicated. The same experiment was repeated three times.

Figure-S5

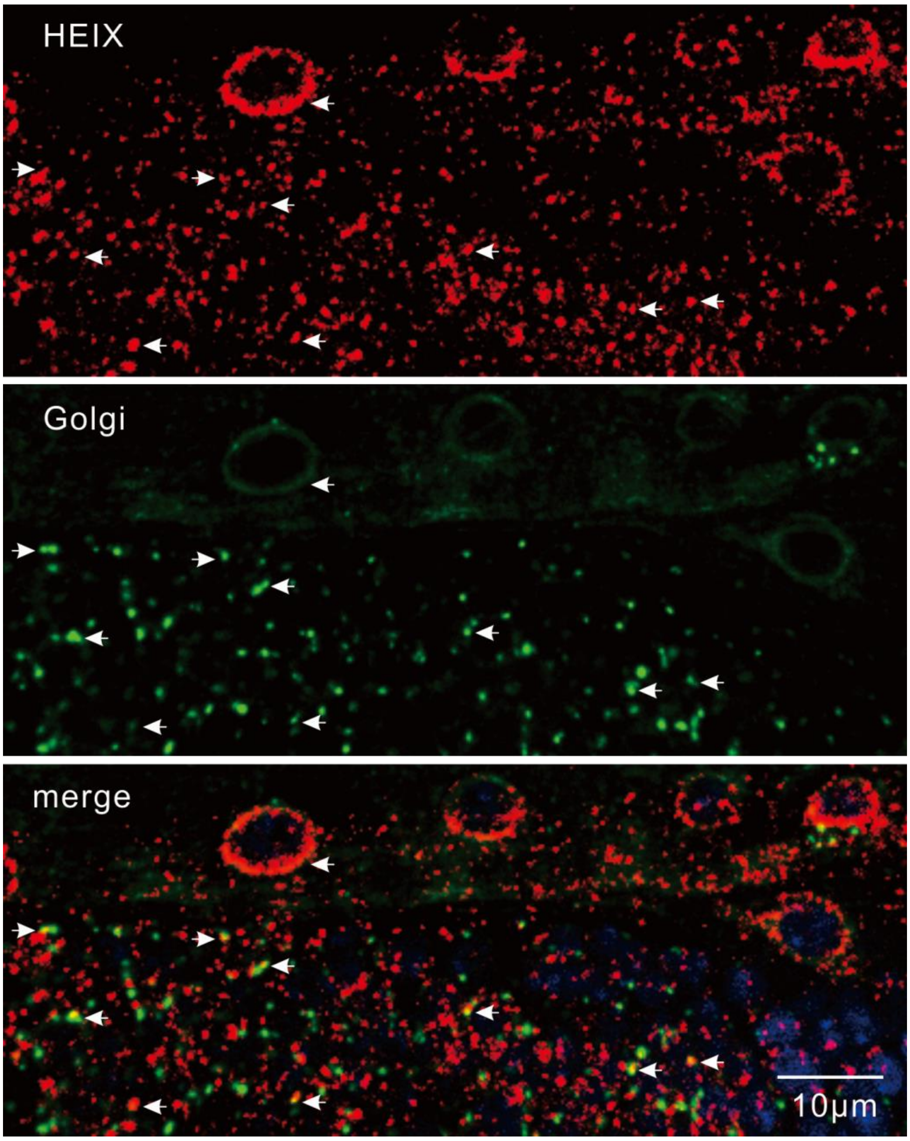

Figure S5 A small portion of Heix is colocalized with Golgi apparatus in blood vessel of wild type larvae, Related to Figure 4

Immunoflourescent images show that a small portion of Heix (red) is colocalized with the Golgi apparatus (green) in cells of blood vessel. Nucleuses were stained with DAPI (blue). The same experiment was repeated three times..

Figure-S6

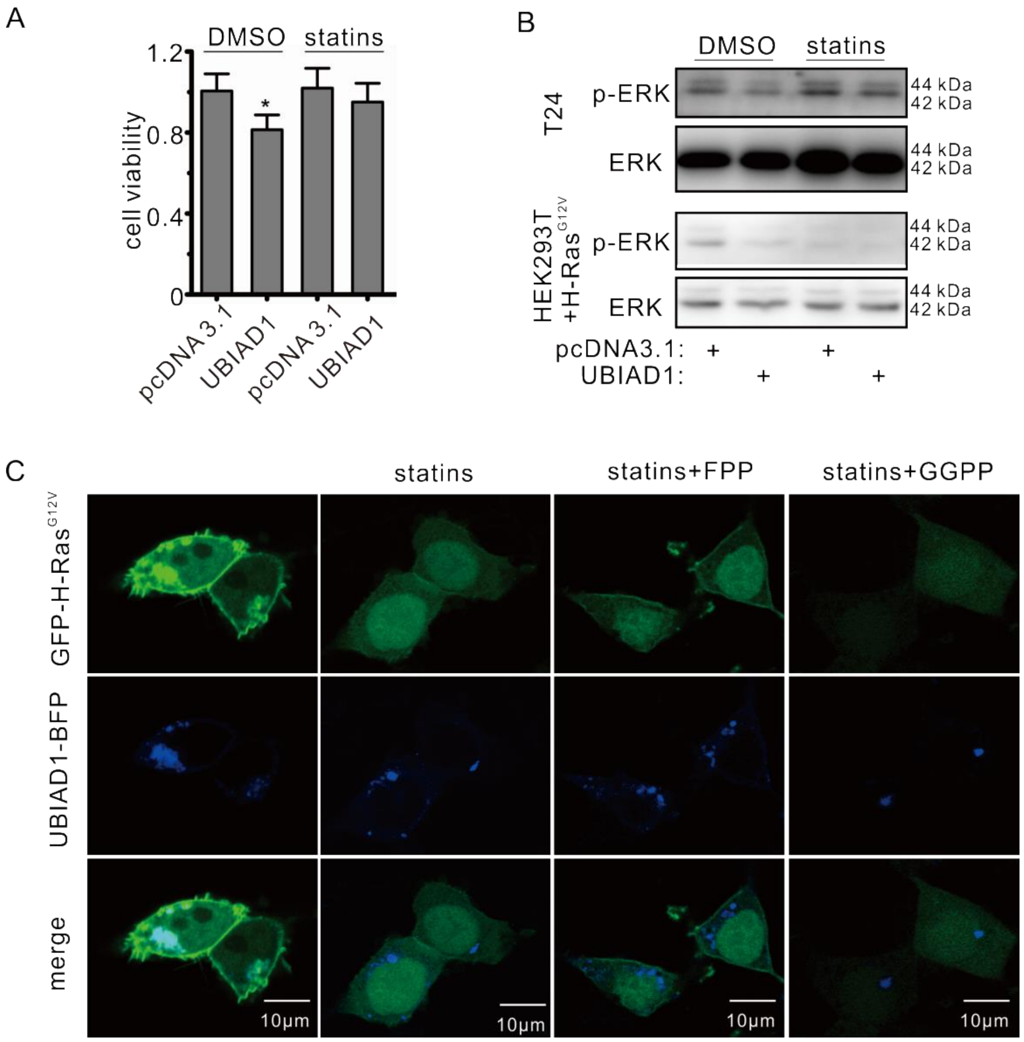

Figure S6 Statins treatment makes both UBIAD1 and Ras misfunction, Related to Figure 6

(A) Statins treatment abolished UBIAD1-induced decrease of T24 cell viability. T24 cells were transfected with pcDNA3.1-UBIAD1. The cells were cultured with or without statins for 24 hours. The cell viability was detected by MTT assay. *$p<0.05$, Student's *t*-test, n=3 experiments.

(B) Statins treatment abolished UBIAD1-induced decrease of ERK phosphorylation. HEK293T or T24 cells were transfected with plasmids as indicated. The cells were cultured with or without statins for 24 hours, followed by WB with antibodies as indicated. The same experiment was repeated three times.

(C) Statins treatment changed the localization of UBIAD1 and H-Ras^{G12V}. HEK293T cells were transfected with GFP-H-Ras^{G12V} and UBIAD1-BFP, followed by 48 hours of

culturing with statins, FPP, GGPP as indicated, and confocal analysis. It showed that UBIAD1 was located with H-Ras^{G12V} in Golgi apparatus. Under statins treatment, UBIAD1 trafficked from the Golgi apparatus to the ER and H-Ras^{G12V} cannot traffic into membrane system. FPP could make H-Ras^{G12V} traffic into membrane but could not influence the localization of UBIAD1. However, GGPP could not make H-Ras^{G12V} traffic into membrane but could make UBIAD1 locate in Golgi apparatus. The same experiment was repeated three times.

Figure-S7

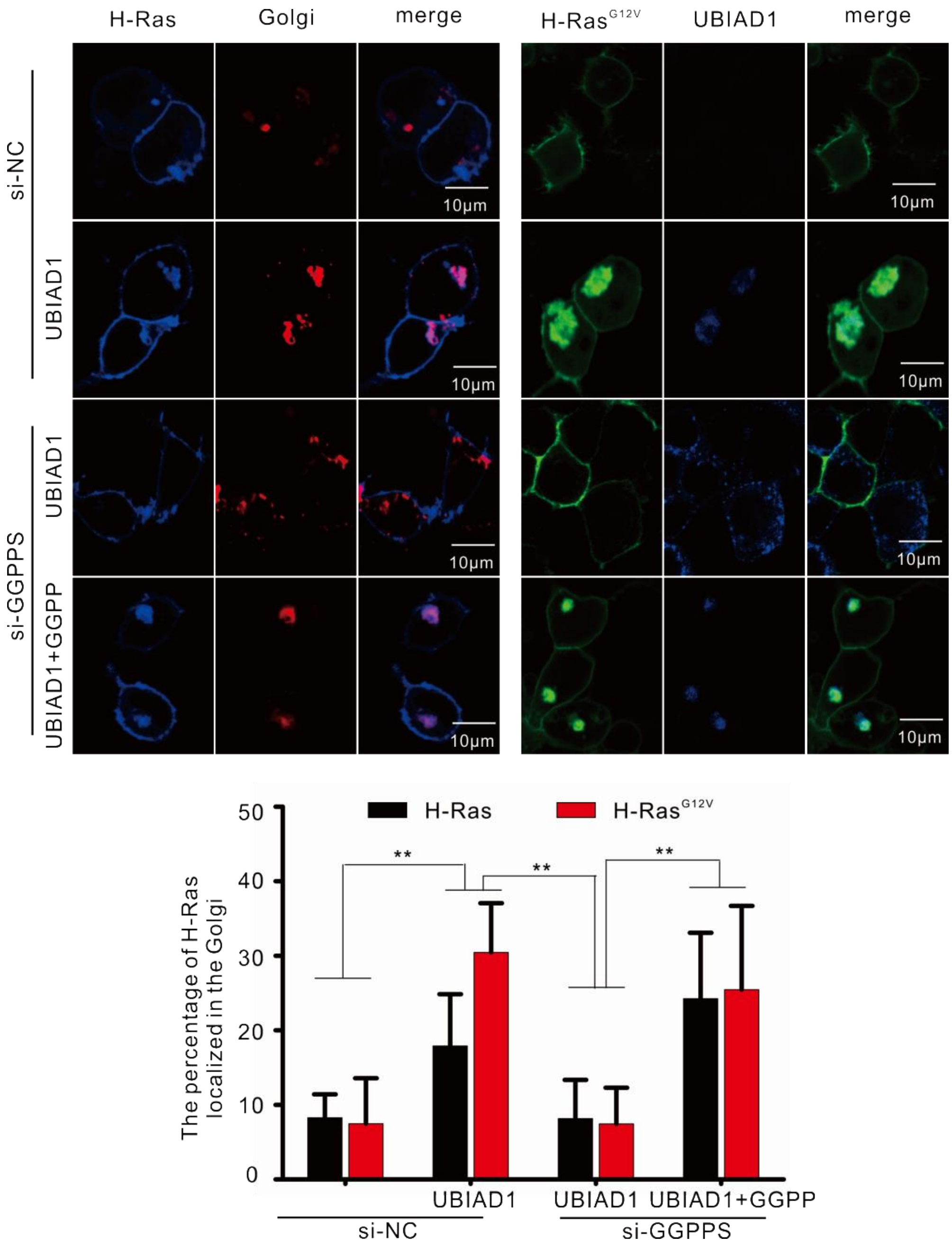

Figure S7 Knocking down GGPPS abolishes UBIAD1-induced H-Ras retention in the Golgi apparatus, Related to Figure 6

HEK293T cells were transfected with si-GGPPS and after 48 hours, the cells were transfected with plasmids as indicated. The cells were cultured with or without GGPP for 24 hours, followed by confocal analysis. The lower panel shows histograms indicating the percentage of Ras localized in the Golgi. **p<0.01 compared to control, (n=3 experiments, each with 100 cells).
